# Application of a modified MSRE-qPCR method for detecting circulating cell-free DNA methylation in cervical cancer

**DOI:** 10.3389/fonc.2026.1759488

**Published:** 2026-05-04

**Authors:** Yaru Liu, Tuping He, Shang Chen, Shenglong Wang, Zhihao Luo, Haixi Liu, Quan Liu, Dixian Luo

**Affiliations:** 1 First Clinical Medical College, Ningxia Medical University, Yinchuan, China; 2 Medical Laboratory Center, the Third Affiliated Hospital (The Affiliated Luohu Hospital), Shenzhen University, Shenzhen, China; 3 Laboratory Medicine Center, Shenzhen Nanshan People’s Hospital, Shenzhen, China; 4 School of Medicine, Hunan University of Chinese Medicine, Changsha, China; 5 Hunan Provincial University Key Laboratory of the Fundamental and Clinical Research on Functional Nucleic Acid, Changsha Medical University, Changsha, China; 6 Department of Prevention and Control, 986th Hospital of Air Force, Xi’an, China; 7 Institute of Pharmacy and Pharmacology, School of Pharmaceutical Science, Hengyang Medical School, Hengyang, China

**Keywords:** cell-free DNA, cervical cancer, cfDNA abundance, DNA methylation, methylation panel, Methylight, MSRE-qPCR

## Abstract

**Background:**

The methylation status of cell-free DNA (cfDNA) shows promise for the clinical detection of cervical cancer and its precursors; however, its measurement in liquid biopsies is hindered by low cfDNA yields and technically demanding protocols.

**Methods:**

We developed an enhanced Methylation-sensitive restriction enzyme-quantitative polymerase chain reaction (MSRE-qPCR) method to create a combinatorial methylation assay with high sensitivity and specificity. Incorporating T4 DNA ligase following MSRE digestion improves the detection of short cfDNA fragments. This T4-assisted system enables methylation analysis of RXFP3-L1, RXFP3-L2, ZNF671, PAX1, and SOX1 for cervical cancer (CESC) evaluation with only 1.5 ng of DNA input; furthermore, lower input is sufficient for assessing the hypermethylated PAX1 gene.

**Results:**

When applied to 24 blood samples from CESC patients and 21 matched tissues, the modified MSRE-qPCR method achieved 87.5% sensitivity and 90.3% specificity using 1.5ng of DNA, outperforming MethyLight without requiring more sensitive quantitative techniques. A methylation panel comprising RXFP3-L1, RXFP3-L2, ZNF671, and SOX1 yielded an AUC of 0.917 (95% CI: 0.830–1.000) in the development set. We constructed a logistic regression model using this gene panel to predict CESC status.

**Conclusion:**

This methodological improvement overcomes the limitation of low cfDNA abundance and provides a practical methylation-based tool for CESC detection.

**Clinical Significance:**

This non-invasive cfDNA methylation assay shows promise as an adjunct for the early detection of cervical cancer. It could also enhance screening adherence relative to conventional cytology, especially in resource-constrained environments.

## Graphical Abstract

**Figure:**
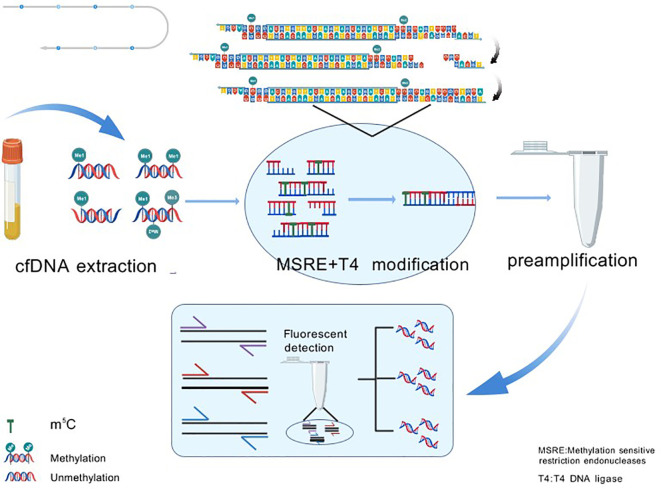
Schematic representation of the modified MSRE-qPCR method for detecting methylated CpG sites in cfDNA. The methodological model of Methylation-Sensitive Restriction Enzyme - Quantitative Polymerase Chain Reaction (MSRE - qPCR) for cell - free DNA (cfDNA) detection is an enzymatic combination application system that integrates PCR amplification and probe signal detection. It encompasses four sequential steps. Initially, cfDNA is extracted from plasma specimens. Subsequently, when the target sequence harbors the recognition site of the methylation - sensitive restriction enzyme, the cfDNA is selectively digested into novel fragments by the MSRE, and T4 DNA ligase ligates the linker to the successfully digested target sequence. Following this, the recombinant template requires pre - amplification. Ultimately, the recombinant cfDNA products are detected via qPCR. Consequently, the amplification specificity and detection sensitivity of each gene can be verified by examining the cycle threshold (Ct) value.

## 1 Introduction

Cervical cancer persists as a predominant malignancy within the female reproductive system, characterized by a high incidence rate and associated mortality (1–4). Patients with advanced - stage cervical cancer may manifest irregular vaginal bleeding, abnormal discharge, pelvic pain, or distant metastasis, which can pose a life - threatening situation. Consequently, early diagnosis is of utmost significance for the prevention and management of cervical cancer. Screening can detect precancerous lesions or carcinoma *in situ*, and timely intervention can achieve cure rates surpassing 90% (5).

Currently, cervical cancer screening encounters substantial challenges. Although human papillomavirus (HPV) testing demonstrates high sensitivity, its lack of specificity results in a large number of false - positive patients being unnecessarily referred for colposcopy (6). Traditional cytology (Pap smear) is restricted by subjective interpretation and low sensitivity for early - stage lesions, and is further exacerbated by poor patient compliance with repeated screenings (7). Cell - Free (cfDNA) methylation testing addresses these limitations. In recent years, cfDNA has emerged as a valuable liquid biopsy biomarker for the early diagnosis and prognostic assessment of cervical cancer. Additionally, persistently positive or rising cfDNA levels before and after treatment are strongly correlated with poorer progression - free survival (PFS) and overall survival (OS) rates, suggesting the potential of using cfDNA to monitor minimal residual disease (MRD) and predict recurrence (8, 9). In conclusion, the severe threat posed by cervical cancer highlights the necessity of early diagnosis. As a non - invasive biomarker that provides real - time insights into tumor dynamics, cfDNA shows considerable potential for overcoming the limitations of traditional screening methods and promoting precision medicine in cervical cancer (10, 11).

We and others have shown that hypermethylated RXFP3 - L1 cg26390889, RXFP3 - L2 cg26986911, cg07832473, cg15408073 (12, 13), ZNF671 cg11977686, cg19246110 (14), SOX1 cg21385666, cg23668285 (15), and PAX1 cg22906273 (16) are simultaneously present in >90% of cervical cancers but absent in the benign cervix, yielding a combined AUC of 0.96 against histological truth. We retrieved the bioinformatics data regarding the correlation between cervical cancer genes and their CpG methylation from the DNA Methylation Interactive Visualization Database(DNMIVD)(see **Supplementary Tables 1**, **2**). However, contemporary workflows fail the clinic: bisulfite conversion can destroy up to 90% of the input DNA and increase costs (17); affinity - enrichment lacks single - CpG resolution (18); and conventional methylation - sensitive restriction enzyme (MSRE) - PCR is rapid but has a limited sensitivity of 1–5% because incomplete digestion and primer noise from short fragments are not eliminated (19).To surmount these constraints, we re-engineered the methylation-sensitive restriction enzyme quantitative polymerase chain reaction (MSRE-qPCR) (20) by incorporating a T4 DNA ligase “second gate.” In our methodology, subsequent to AciI/HhaI cleavage that specifically targets unmethylated CCGC/GCGC sites, intact hypermethylated molecules are ligated to a universal hairpin adapter. Subsequently, quantitative polymerase chain reaction (qPCR) employing an adapter-specific forward primer and a gene-specific reverse primer is implemented to ensure that only full-length, methylation-protected templates are amplified. This approach facilitates the multiplexed tracking of RXFP3, ZNF671, SOX1, and PAX1 methylation in cervical cell-free DNA (cfDNA), rendering it applicable for large-scale screening, therapy response monitoring, and minimal residual disease (MRD) surveillance. This modified MSRE-qPCR (MSRE-T4L-qPCR) method (**Graphical Abstract**) was validated via a 0.01% methylation gradient dilution, enzyme digestion–ligation dual-blocking, and a background cycle threshold (Ct) delay of ≥6 cycles, demonstrating significantly higher sensitivity and specificity compared to conventional MSRE-qPCR.

To assess the clinical performance, we conducted an external validation utilizing a three-tier sample strategy. Initially, blinded testing was performed on plasma cell-free DNA (cfDNA) from 24 cervical cancer patients prior to treatment, with matched surgical tissue DNA from 21 patients serving as the gold standard. Plasma from 12 healthy females was employed to establish the baseline noise. The methylation sites RXFP3-L1, RXFP3-L2, ZNF671, and SOX1 were selected as detection targets, forming a four-gene panel. At the individual gene level, receiver operating characteristic (ROC) curves were constructed, and the area under the curve (AUC) was calculated. The tissue–plasma paired analysis yielded single-gene AUC values ranging from 0.81 to 0.88, whereas the four-gene logistic regression model achieved an AUC of 0.917 (95% confidence interval [CI]: 0.830–1.000), with 87.5% sensitivity, 90.3% specificity, 84.8% positive predictive value (PPV), and 92.1% negative predictive value (NPV). These results corroborate that the modified MSRE-qPCR accomplishes its intended detection objective. Using independent samples, we further established quantitative interpretation criteria, resulting in a robust cfDNA methylation detection model that requires only 1.5 ng of input sample and completes the entire process within 3 h.

## 2 Materials and methods

### 2.1 Screening of methylated genes and CpG sites in cervical cancer

Artificial cell-free DNA (cfDNA) templates for validating the efficiency of methylation-sensitive restriction enzymes (MSREs) and T4 DNA ligase were synthesized by Beijing Ruibo Xingke Biotechnology Co., Ltd. The synthesized single-stranded DNA necessitated specific processing to form double strands. All cervical cancer hotspot genes and CpG sites were retrieved from the DNMIVD (http://119.3.41.228/dnmivd/index/). The annealing reaction for double-strand formation employed a system comprising 22.5 μL of 10 μM upstream sequence, 22.5 μL of 10 μM downstream sequence, and 5 μL of 10×rCutSmart Buffer. The annealing protocol entailed initial denaturation at 98 °C, followed by cooling to 25 °C at a rate of 0.1 °C/s, and final storage at 4 °C. Adapter sequences were analogously hybridized into double strands using identical reaction conditions and thermal cycling parameters. Methylated and non - methylated templates were mixed at ratios of 0:10, 2:8, 4:6, 6:4, 8:2, and 10:0, with the total template concentration maintained at a constant level of 0.001 ng/μL. All prepared cfDNA templates were stored at −20 °C.

### 2.2 Patient cohort and sample collection

This was a cross - sectional observational study. Longitudinal sampling was not conducted since the primary aim was to assess diagnostic accuracy. This study was approved by the Ethics Committee of Shenzhen Nanshan People’s Hospital (Approval No.: 20190517). Participants were recruited from Shenzhen Nanshan People’s Hospital and Chenzhou First People’s Hospital. A total of 36 blood samples were collected, including 24 from cervical cancer patients and 12 from healthy volunteers, along with 21 paired pathological tissue specimens. For inclusion, participants were female, with a mean age of 45.6 years, diagnosed with cervical cancer through histopathology, and provided an adequate amount of peripheral blood (**Supplementary Tables 3**, **4**). Patients with insufficient blood volume, other severe autoimmune diseases, or without cervical histopathological results were excluded. All participants provided written informed consent.

Serum (5 mL) or plasma (3 mL) was collected from each participant using serum separation tubes containing clot activator and separation gel or EDTA anticoagulant tubes, respectively. The samples were stored at 4 °C and processed within 4 hours. For plasma preparation, whole blood was centrifuged at 1600 × g and 4 °C for 15 minutes, and the plasma layer was subsequently recentrifuged under identical conditions to remove residual cellular material. Processed plasma samples were stored at −80 °C for future use. Serum was obtained by allowing blood to clot at room temperature for 30–60 minutes, followed by centrifugation at 1500 × g for 10 minutes under ambient conditions. Serum samples were also stored at −80 °C. Tumor tissues obtained via surgical resection were also preserved at −80 °C for subsequent analysis.

A modified MSRE - qPCR procedure was employed for the cfDNA artificial template validation experiments. Synthetic templates containing specific methylation sites and non - methylated controls, provided by Guangzhou Ruibo Co., Ltd., were processed in accordance with the manufacturer’s instructions and served as cfDNA templates to evaluate the selected sequences, methylation sites, and overall experimental feasibility.

### 2.3 cfDNA extraction and quantification

Circulating cell-free DNA (cfDNA) was isolated from 1–3 mL of cell-free plasma utilizing the QIAamp ccfDNA/RNA Kit (Qiagen, Cat. No. 55184) in accordance with the manufacturer’s guidelines. Specifically, the plasma was subjected to treatment with a lysis buffer containing proteinase, followed by isopropanol precipitation, column-based purification, and elution in 100 μL of RNase-free water. The isolated cfDNA was quantified via a Nanodrop dsDNA spectrophotometer. (For detailed procedures, refer to the instructions).

### 2.4 gDNA extraction and quantification

Genomic DNA was isolated from 25 mg of pathological tissue utilizing the Roche High Pure PCR Template Preparation Kit (Roche, Cat. No. 11796828001) in accordance with the manufacturer’s protocols. Specifically, the tissue was digested with Proteinase K at 55 °C for 1 hour, followed by binding to a purification column, washing, and elution in 200 μL of buffer. The extracted DNA was quantified via a Nanodrop dsDNA spectrophotometer and stored at −20 °C for subsequent analyses. (Detailed steps can be referred to in the instructions).

### 2.5 MSRE digestion coupled with T4 DNA ligation

The AciI/HhaI digestion reaction mixture was formulated by combining 1 μL of 10×rCutSmart Buffer, 1 μL of AciI/HhaI (New England Biolabs, Catalog Number: R0551L/R0139L), 20 ng of cell - free DNA (cfDNA) or genomic DNA (gDNA), and diethylpyrocarbonate (DEPC) water to achieve a final volume of 10 μL. This reaction mixture was incubated at 37 °C for 60 minutes and subsequently heat - inactivated at 65 °C for 25 minutes. The digested products were subjected to separation through electrophoresis on a 2% tris - acetate - EDTA (TAE) agarose gel at 120 V for 30 minutes, and the gel was visualized using a gel imaging system (Thermo Fisher Scientific).

The T4 DNA ligation reaction mixture consisted of 2 μL of 10×T4 Ligation Buffer, 2 μL of T4 DNA Ligase (New England Biolabs, Catalog Number: M0202V), 6 ng of methyl - sensitive restriction enzyme (MSRE) - digested cfDNA or tissue - derived DNA (tiDNA) product, and DEPC water to reach a final volume of 20 μL. Ligation was carried out at 25 °C for 60 minutes, following which the enzyme was inactivated by heating at 65 °C for 25 minutes. The ligated products were electrophoresed on a 2% TAE agarose gel at 120 V for 30 minutes and analyzed using the identical imaging system.

### 2.6 Optimized MSRE-qPCR for low-input cfDNA methylation detection

The specificity of primers and probes was ensured through a comprehensive validation process. Initially, an in silico specificity analysis was performed via BLAST searches against the human genome, and no significant off - target binding was identified. During the design stage, the cervical cancer methylation genes involved in this study were initially analyzed. High - level methylation markers and their associated CpG sites were identified from the DNMIVD. Sequence fragments encompassing the target loci were retrieved using the NCBI BLAST tool. Subsequently, primers and probes were designed using SnapGene software in accordance with sequence characteristics and MSRE cleavage site features. The designed oligonucleotides were re - assessed by BLAST to confirm the absence of interfering sequences.

Primers and TaqMan probes for GAPDH, PAX1, SOX1, RXFP3 - L1, RXFP3 - L2, and ZNF671 were designed based on CpG site characteristics and standard design principles. Their sequences are as follows: PAX1—forward primer: 5′ - CCA AAG GGC CGC AGT GAC - 3′, reverse primer: 5′ - GAC GTG TCC TCC ACG TCA ATC TC - 3′, probe: FAM - 5′ - CAC GCC GGA GAC GCG C - 3′ - BHQ1; SOX1—forward primer: 5′ - GGC TCT GAC GTT ACC TTG C - 3′, reverse primer: 5′ - CTT CCT CCC TCC CTC TGG - 3′, probe: FAM - 5′ - CAG GTG GAA GGC GCC CCG C - 3′ - BHQ1; RXFP3 - L1—forward primer: 5′ - CCT CAT CCA AGC AGT CCC - 3′, reverse primer: 5′ - GAA TGC GAT CTT GCG CTC - 3′, probe: FAM - 5′ - GGG CCG CTC GCT CCC - 3′ - BHQ1; RXFP3 - L2—forward primer: 5′ - GCG ATC TTG CGC GCC CTT G - 3′, reverse primer: 5′ - CGT CTC TCC GCG GTT GTC - 3′, probe: FAM - 5′ - GCC AGC GGC TCT CAC C - 3′ - BHQ1; and ZNF671—forward primer: 5′ - GAA TGC GAT CTT GCG CGC TTT C - 3′, reverse primer: 5′ - GTA GCG GAC ATT TTG TTT CTG T - 3′, probe: FAM - 5′ - GTG GGC CGC AGG T - 3′ - BHQ1 (see **Supplementary Table 5**).

The selection of AciI and HhaI was predicated on their capacity to differentially cleave unmethylated CpG sites within the promoter regions of genes such as PAX1, with precedence accorded to genes that, as per data analysis from DNMIVD, display a higher frequency of methylation in cervical cancer. The optimized MSRE - qPCR procedure for DNA methylation detection consisted of four steps (**Graphical Abstract**): digestion of cfDNA with methylation - sensitive restriction enzymes (MSREs) to cleave unmethylated CpG sites and diminish background; ligation utilizing T4 DNA Ligase to attach MSRE - specific adapters and form recombinant cfDNA; pre - amplification with the Q5 system in a 20 - μL reaction ((2×Q5 High - Fidelity Master Mix, 1.5 ng T4 - ligated product, 10 μM forward and reverse primers, and DEPC water) with cycling conditions of (98 °C for 30 s; 25 cycles of 98 °C for 5 s, 50 °C for 10 s, and 72 °C for 20 s; final extension at 72 °C for 2 min); and qPCR detection employing TaqMan probes in a mixture of 10 μL 2×ChamQ Geno SNP Probe Master Mix, 10 μM forward and reverse primers, 10 μM probe, and 1 μL pre - amplified product, with cycling conditions of 95 °C for 30 s; 45 cycles of 95 °C for 10 s and 60 °C for 30 s; and a final step at 60 °C for 30 s. Ct values were obtained on a 7500 Fast Real - Time PCR System (Thermo Fisher Scientific, USA) using software v2.3 with the threshold set at 0.12. The SLAN^®^ - 96S (Hongshi, Shanghai) platform with software v8.8.2 was also utilized under identical threshold settings.

To assess the sensitivity of the enhanced MSRE - qPCR assay, methylated and unmethylated cfDNA templates were mixed in serial ratios. The total template concentration was kept at 0.001 ng/μL, with the methylated fraction ranging from 0.001 ng to 0.01 ng and the unmethylated portion decreasing proportionally from 0.01 ng to 0.001 ng. Amplification Ct values were recorded and graphed, and PCR products were verified via agarose gel electrophoresis.

### 2.7 Bisulfite conversion and quality control

The bisulfite conversion of cell-free DNA (cfDNA) was carried out using the methylation detection sample pre-treatment kit (Yaneng, Shenzhen, China) in accordance with the manufacturer’s specifications. Specifically, a maximum of 200 ng of DNA was denatured at 98 °C and subsequently treated with sodium bisulfite at 64 °C, with a total conversion duration of 120 min (comprising three cycles of 40 min each). The converted DNA was purified via a column-based system utilizing binding, wash, and desulfonation buffers, and then eluted in 20 μL of elution buffer. The final bisulfite-converted DNA was stored at –20 °C for short-term utilization or –80 °C for long-term conservation. (Refer to the instructions for detailed procedures).

### 2.8 MethyLight assay

MethyLight analysis was conducted utilizing the ABI 7500 Real - Time PCR System (Applied Biosystems, Foster City, CA), employing primers and probes specific to bisulfite - converted DNA. The reaction was targeted at the PAX1, SOX1, HAS1, and ACTB (internal control) genes. The fluorophore - quencher pairs were configured as follows: PAX1 (FAM - BHQ1), SOX1 (VIC - BHQ1), HAS1 (CY5 - BHQ3), and ACTB (Texas Red - BHQ2). The PCR cycling conditions entailed an initial denaturation at 95 °C for 15 min, succeeded by 45 cycles of 95 °C for 15 s and 58 °C for 45 s. Methylation quantification was predicated on ΔCt [Ct(target gene) – Ct(ACTB)], and the interpretation criteria were elaborated in **Supplementary Tables 11**, **12**. (Detailed steps can be referred to in the instructions).

### 2.9 Statistics and reproducibility

No statistical methods were employed to pre - determine the sample size. The experiments were carried out without randomization or blinding. Data analyses were executed using GraphPad Prism 10.1.2 (La Jolla, CA, USA), and the results were presented as mean ± standard deviation ((SD). A *p* - value less than 0.05 was regarded as indicating statistical significance. All experiments were independently replicated three times. Parametric tests (e.g., t - test) or non - parametric tests (e.g., Mann–Whitney test) were selected based on data normality. Multigroup comparisons were conducted via one - way ANOVA or the corresponding non - parametric test. Two - tailed *p* - values were utilized throughout. Figures were prepared with Adobe Illustrator. The relative fluorescence intensity of each sample relative to the negative control was computed using the following formulas: sensitivity = true positive/(true positive + false negative); specificity = true negative/(true negative + false positive); and Youden index (J) = sensitivity + specificity − 1. The cutoff value was defined as the detection threshold corresponding to the maximum Youden index.

A three - gene, four - locus logistic regression model was developed using SPSS 27.0 (SPSS Inc., Chicago, IL, USA). Using the “actual status” as the gold standard (positive = 1, negative = 0), non - parametric ROC curves were generated in accordance with the methylation levels (β values, ranging from 0 to 1) of four loci: ZNF671, RXFP3 - L1, RXFP3 - L2, and SOX1. The null hypothesis (H_0_) posited that the true AUC was equal to 0.5, indicating no discriminative ability. The alternative hypothesis ((H_1_) proposed that the true AUC exceeded 0.5. A significance level of α = 0.05 was adopted, and all tests were two - tailed.

## 3 Results

### 3.1 Bioinformatic screening of candidate methylation markers for cervical cancer using the DNMIVD database

**Figure 1:**
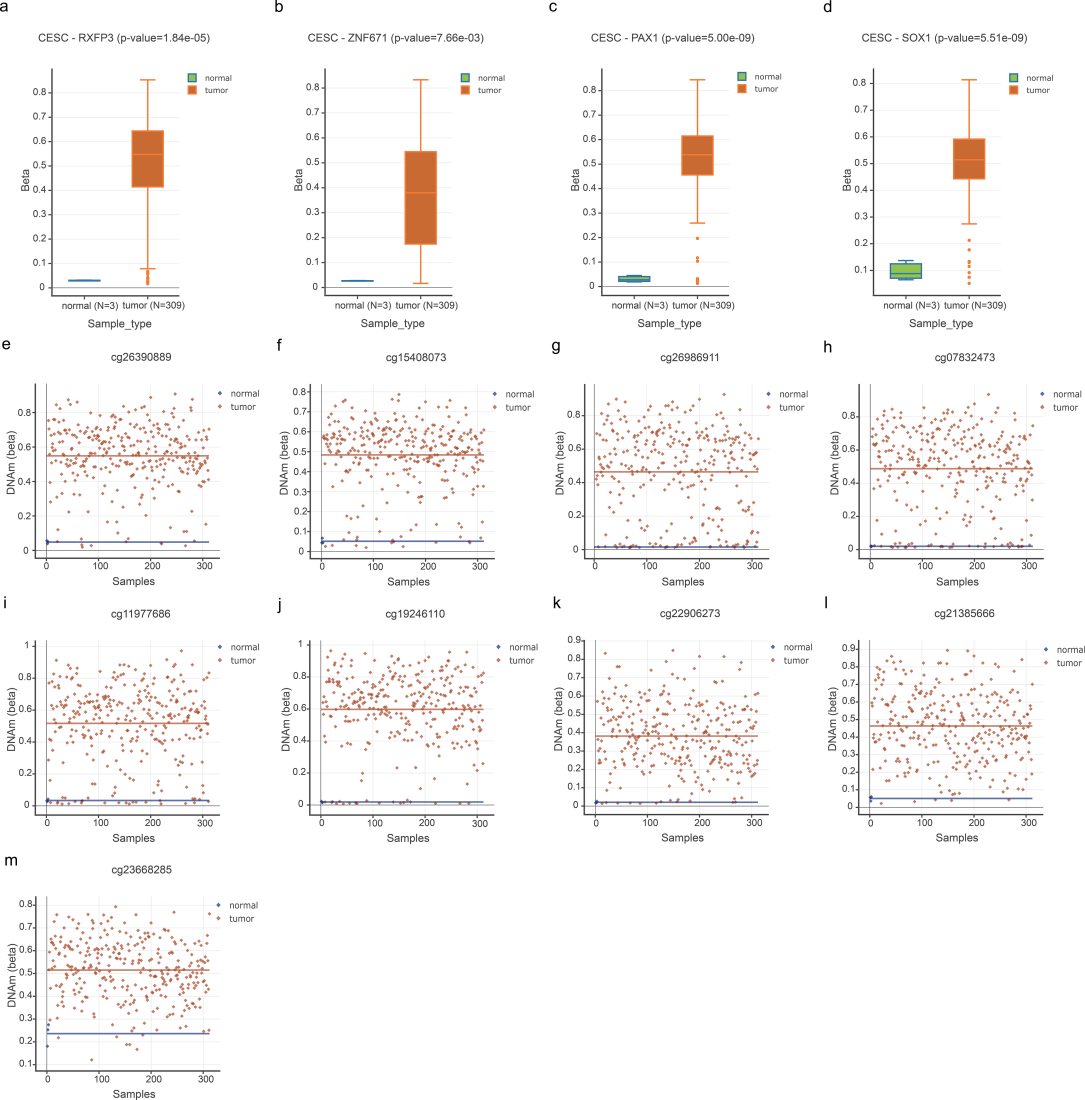
Differential DNA methylation in CESC. **(A–D)** Boxplots of DNA methylation for RXFP3, ZNF671, PAX1, and SOX1 in normal (n = 3) and tumor (n = 309) samples (two-tailed Student’s t test; P-values: 1.84e-05, 7.66e-03, 5.00e-09, 5.51e-09). **(E–M)** Scatter plots of DNAm levels for nine CpG sites, showing elevated methylation in tumor samples (all P < 0.0001).Boxes show IQR with median; whiskers extend to 1.5×IQR. ****P < 0.0001, **P < 0.01. Horizontal lines denote median values; the Y - axis is labeled “DNAm (beta)”, and the X - axis presents a comparison between “Normal (n = 3)” and “Cancer (n = 309)”. The color scale ranges from blue (representing normal samples) to red (representing tumor samples).

To identify the optimal methylation markers for cervical cancer, we initially conducted an analysis of four candidate genes (RXFP3, ZNF671, PAX1, and SOX1) using the DNMIVD. A comparison of methylation β - values between 306 cervical cancer patients and three healthy controls unveiled distinct differential methylation patterns. **Figure 1** depicts these results: (A - D) Box plots present the methylation levels of RXFP3 (A), ZNF671 (B), PAX1 (C), and SOX1 (D) in the cervical squamous cell carcinoma (CESC) and normal groups; (E - H) Scatter plots illustrate the distribution of eight CpG loci for RXFP3 (E), ZNF671 (F), PAX1 (G), and SOX1 (H). RXFP3 exhibited significantly increased methylation in cervical cancer (p < 0.05, **Figures 1A, E**), as did PAX1 (p < 0.05, **Figures 1C, G**) and SOX1 (p < 0.05, **Figures 1D, H**). In contrast, ZNF671 did not display significant differential methylation (**Figures 1B, F**), which is consistent with its limited discriminatory capacity.

Validation within the DNMIVD cohort corroborated that PAX1, SOX1, and RXFP3 exhibited significant differential methylation across groups, while ZNF671 did not (**Supplementary Table 1**). These three genes underwent hypermethylation in cervical cancer, and the magnitude of methylation alteration was ranked as SOX1 > PAX1 > RXFP3. Among the promoter CpG sites analyzed, the most differentially methylated sites, ranked by adjusted p - value, were as follows: cg26390889 (RXFP3 - L1) > cg15408073 (RXFP3 - L2) > cg19246110 (ZNF671) > cg23668285 (SOX1) > cg21385666 (SOX1). When ranked by mean β - value, the sequence was: cg19246110 > cg26390889 > cg11977686 > cg23668285 > cg07832473 > cg15408073 > cg26986911 > cg21385666 > cg22906273 (**Supplementary Table 2**). These bioinformatics findings directly informed our selection of specific CpG loci for subsequent assay development: cg22906273 (PAX1), cg21385666/cg23668285 (SOX1), cg26390889 (RXFP3 - L1), and cg26986911/cg07832473/cg15408073 (RXFP3 - L2), with PAX1, SOX1, and RXFP3 being prioritized as the three validated hypermethylated biomarkers in cervical cancer.

### 3.2 Technical development and optimization of the MSRE-qPCR assay

Based on these candidate markers, a modified multiplexed site-specific restriction enzyme quantitative polymerase chain reaction (MSRE-qPCR) assay was developed. This assay incorporated T4 DNA ligase-mediated adapter ligation to improve the detection efficiency of fragmented cell-free DNA (cfDNA) (**Graphical Abstract**). In the assay design, AciI was utilized for RXFP3-L1, RXFP3-L2, and ZNF671, while HhaI was employed for PAX1 and SOX1. The optimal enzyme concentration was determined to be 5 units per 20 ng of cfDNA. A crucial innovation of this approach is the T4 DNA ligase-mediated adapter ligation, which facilitates the efficient detection of methylated CpG sites in highly fragmented cfDNA.

**Figure 2:**
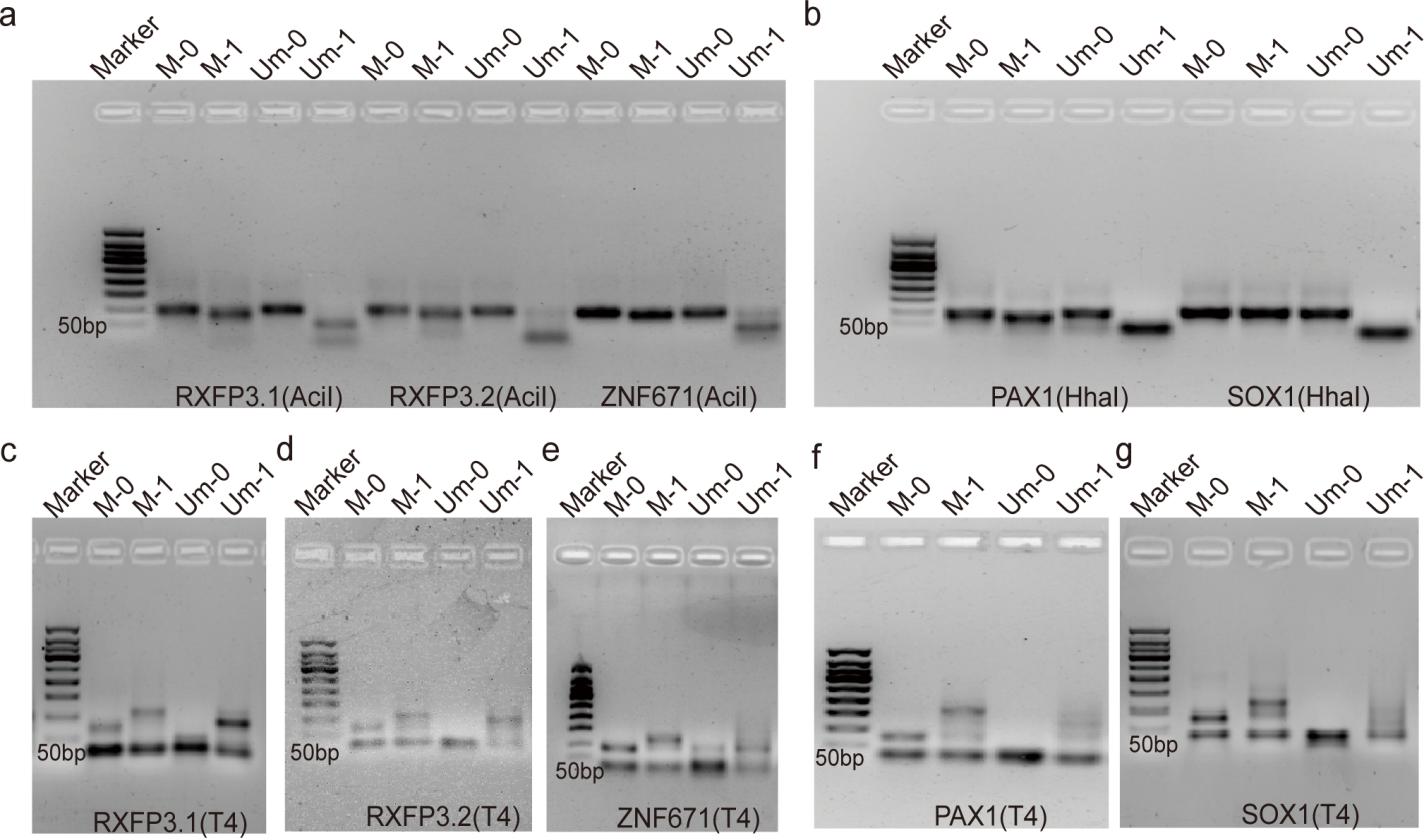
Combination enzyme treatment enriched the sequence template DNA electropherograms of genes containing specific methylated CpG sites. “M” indicates methylation and “Um” indicates non-methylation. “0” shows that the template was not digested due to no MSRE(T4), serving as a negative control group, however, “1” indicates a template acting by adding a specific MSRE(T4); **(A)** The RXFP3-L1, RXFP3-L2, and ZNF671 gene templates were digested with AciI and analyzed by electrophoresis; **(B)** The PAX1 and SOX1 gene templates were similarly treated with HhaI and subjected to electrophoretic analysis; **(C-G)** Electrophoregram of T4 DNA ligase-catalytically enriched ligated templates with recombination modified templates with designed adaptor junctions, these genes are RXFP3-L1, RXFP3-L2, ZNF671, PAX1 and SOX1.

During the optimization process, the enzyme digestion efficiency and adapter ligation performance were systematically evaluated. Gel electrophoresis analysis indicated that RXFP3-L2, which contains three methylated CpG sites, demonstrated superior detection efficiency compared to RXFP3-L1 ((with a single CpG site), as manifested by lower cycle threshold (Ct) values and stronger fluorescence signals (**Supplementary Figures 1A, B**). **Figure 2** depicts the enzymatic processing of target sequences: AciI digestion of RXFP3-L1, RXFP3-L2, and ZNF671 (**Figure 2A**), HhaI digestion of PAX1 and SOX1 (**Figure 2B**), and T4 DNA ligase-catalyzed adapter ligation for all five target sequences (**Figures 2C–G**). The higher recombinant template conversion rate of RXFP3-L2 compared to RXFP3-L1 confirmed that the density of CpG sites is correlated with detection sensitivity.

Analytical validation through the utilization of synthetic templates further verified a linear detection range spanning from a 10 - to 10^5^ - fold dilution (**Supplementary Figure 1A**). It also demonstrated complete discrimination between methylated and unmethylated sequences at mixing ratios ranging from 0:10 to 10:0 (**Supplementary Figures 1B**, **2A, B**), thereby validating the robustness of the modified assay for subsequent clinical validation.

### 3.3 Diagnostic performance of the methylation panel in clinical samples

Armed with the optimized assay, we embarked on an evaluation of its clinical performance by utilizing blood samples from 24 cervical cancer patients, 21 matched tissue samples, and 12 healthy controls (**Supplementary Tables 3**, **4**). Detection was carried out using primers and TaqMan probes enumerated in **Supplementary Table 5**, and the results are presented in **Supplementary Tables 6**-**8**. The concentration of circulating cell - free DNA (cfDNA) extracted from the 24 cervical squamous cell carcinoma (CESC) blood samples spanned from 365.56 ng/mL to 2453.5 ng/mL, with a median value of 670 ng/mL (**Supplementary Figure 3**).

**Figure 3:**
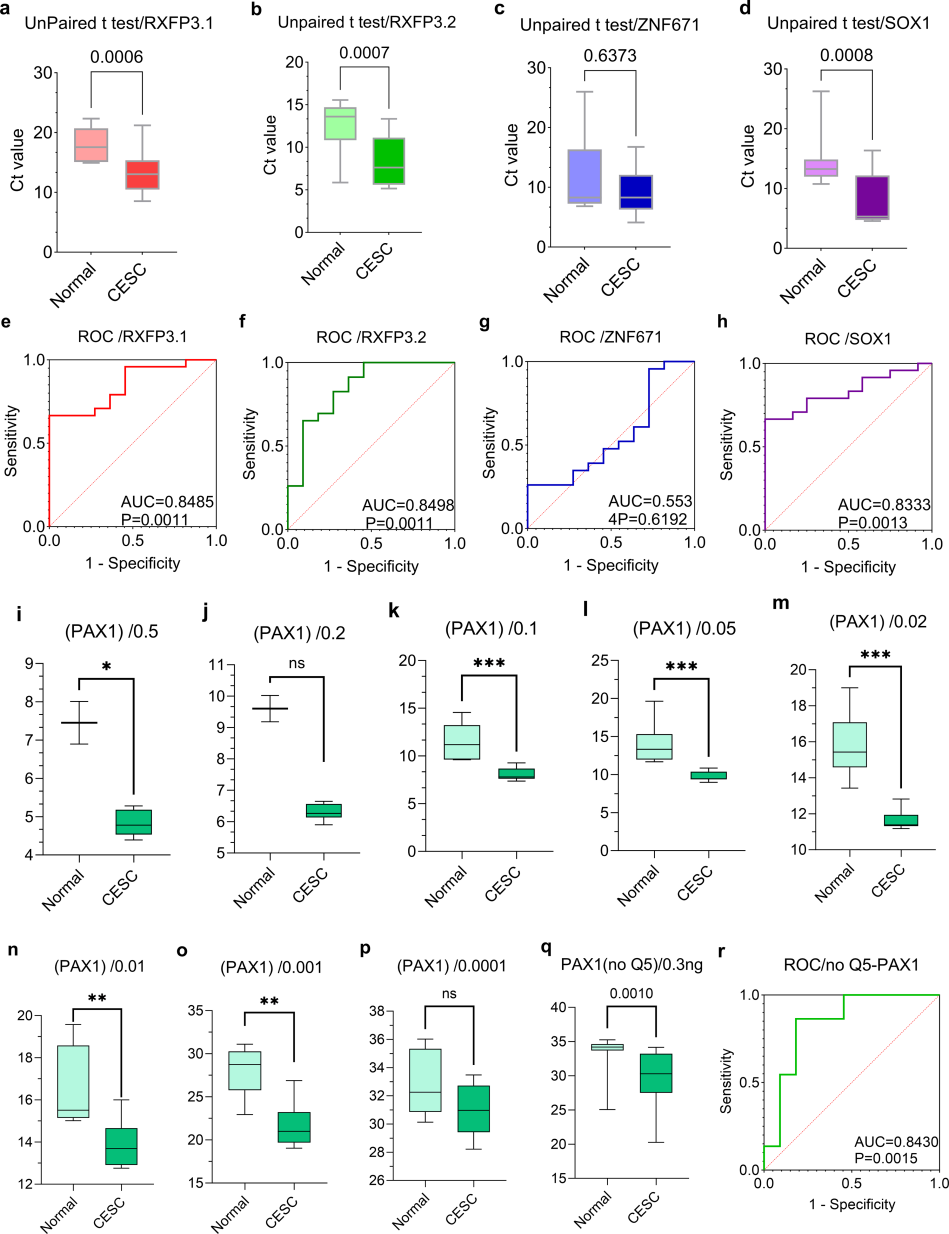
**(A)** The cycle threshold (Ct) values of the RXFP3 - L1 gene were determined in patients with cervical cancer and healthy controls using the MSRE - T4L - quantitative polymerase chain reaction (qPCR) method. **(B)** The Ct values of the RXFP3 - L2 gene were measured in cervical cancer patients and healthy controls via the MSRE - T4L - qPCR method. **(C)** The Ct values of the ZNF671 gene were ascertained in cervical cancer patients and healthy controls by means of the MSRE - T4L - qPCR method. **(D)** The Ct values of the SOX1 gene were quantified in cervical cancer patients and healthy controls using the MSRE - T4L - qPCR method. **(E)** The receiver operating characteristic (ROC) curve for RXFP3 - L1 was generated based on the data from group a. **(F)** The ROC curve for RXFP3 - L2 was derived from the data in group b. **(G)** The ROC curve for ZNF671 was constructed using the data from group c. **(H)** The ROC curve for SOX1 was obtained from the data in group d. **(I-P)** The MSRE - T4L - qPCR method was employed to measure and compare the Ct values of the PAX1 gene across serial dilution ratios (50%, 20%, 10%, 5%, 2%, 1%, 0.1%, and 0.01%) in cervical cancer patients and healthy controls. **(Q)** The PAX1 gene was directly assayed without pre - amplification using the MSRE - T4L - qPCR method, and the resultant Ct values were compared between the cervical cancer group and the normal control group. **(R)** The ROC curve for PAX1 was derived from the data in group q.

The four - marker methylation panel attained a sensitivity of 87.5% (95% confidence interval (CI): 83.3 - 91.7%), a specificity of 90.3% (95% CI: 88.9 - 92.1%), and an area under the curve (AUC) of 0.917 (95% CI: 0.830 - 1.000) in differentiating cervical cancer patients from healthy controls. Among the 24 patient tissue samples, ZNF671 methylation was undetectable in one case; otherwise, all five gene sequences were consistently detected in cfDNA. The method effectively evaluated the methylation levels of RXFP3 - L1, RXFP3 - L2, ZNF671, and SOX1 in blood samples, uncovering significant disparities between patients and controls (**Figures 3A–H**). RXFP3 - L1 displayed significant inter - group differences (p = 0.0006, **Figure 3A**), with an AUC of 0.8485 (p = 0.0011, **Figure 3E**). RXFP3 - L2 demonstrated pronounced differential methylation (p = 0.0007, **Figure 3B**), with an AUC of 0.8498 (p = 0.0011, **Figure 3F**). ZNF671 failed to distinguish between groups (p > 0.05, **Figures 3C, G**). SOX1 exhibited marked differential methylation (p = 0.0008, **Figure 3D**), with a receiver operating characteristic (ROC) AUC of 0.8333 (p = 0.0013, **Figure 3H**).

Notably, the PAX1 gene sequences in cell - free DNA (cfDNA) generated detectable fluorescence signals even in the absence of Q5 pre - amplification (**Figures 3I–R**). Serial dilution experiments demonstrated significant disparities at moderate dilutions (1:1, p = 0.0444, **Figure 3I**; 1:2, p = 0.0364, **Figure 3J**), highly significant disparities at intermediate dilutions (1:10, p = 0.0004, **Figure 3K**; 1:20, p = 0.0007, **Figure 3L**; 1:50, p = 0.0007, **Figure 3M**), and significant disparities at high dilutions (1:100, p = 0.007, **Figure 3N**; 1:1000, p = 0.0047, **Figure 3O**), while there was no significant difference at extreme dilution (1:10000, **Figure 3P**). PAX1 methylation was detectable without pre - amplification, exhibiting significant differences between groups (p = 0.001, **Figure 3Q**), with an area under the curve (AUC) of 0.8430 (p = 0.0015, **Figure 3R**).

### 3.4 Method comparison: modified vs. conventional MSRE-qPCR and tissue-plasma correlation

**Figure 4:**
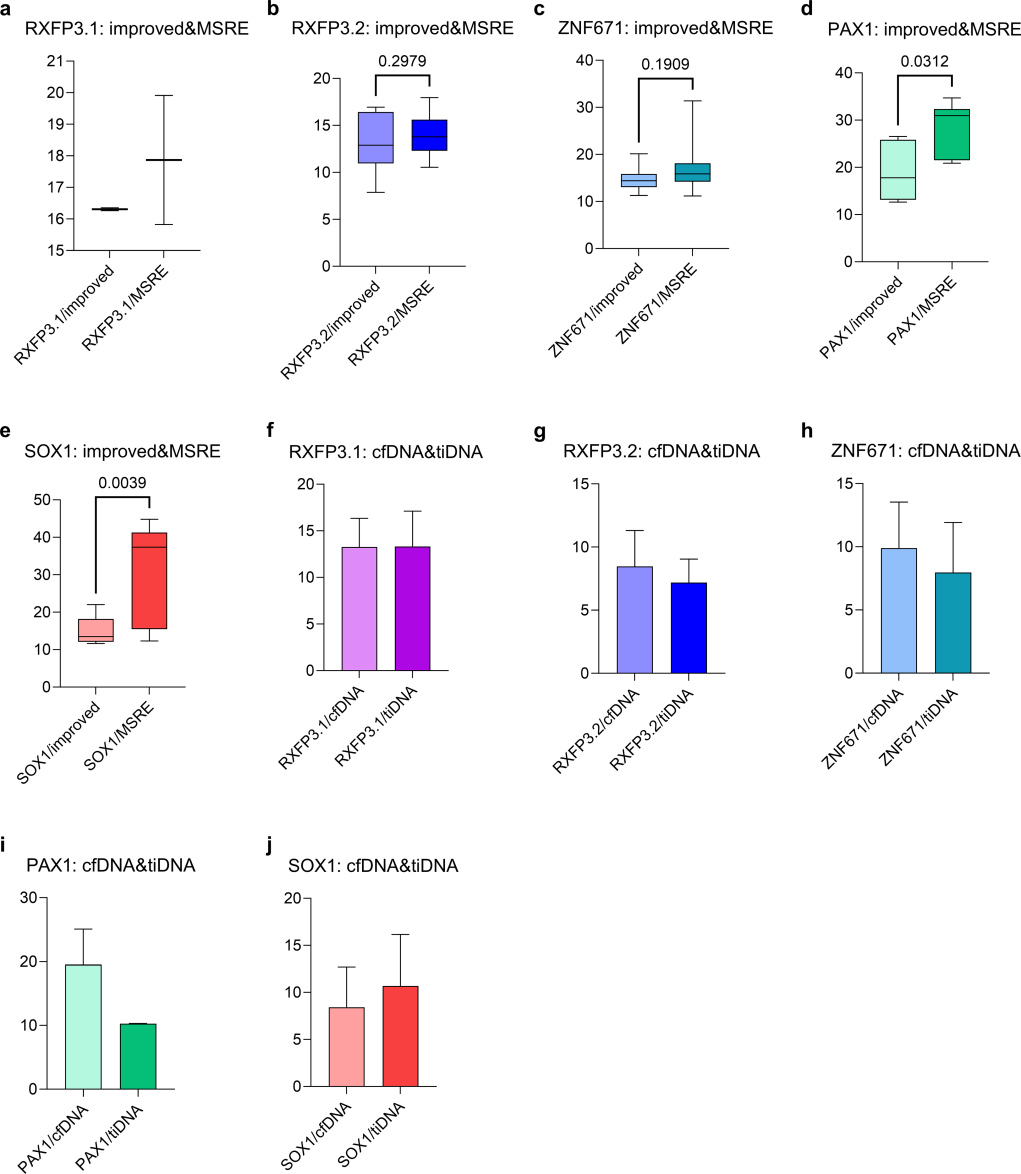
**(A-E)** A comparison of Ct values for RXFP3-L1, RXFP3-L2, ZNF671, PAX1, SOX1 methylation detection reveals differences between the MSRE-4TL-qPCR and MSRE-qPCR methods; **(F-J)** RXFP3-L1, RXFP3-L2, ZNF671, PAX1, and SOX1 were concurrently quantified in paired cfDNA and tissue DNA samples via MSRE-4TL-qPCR, and their respective Ct values were compared.

To validate the technical improvements of our approach, the modified MSRE - qPCR (incorporating T4 DNA ligase) was directly compared with the conventional protocol lacking ligation, utilizing identical primer - probe sets. **Figures 4A-E** depict these comparisons. For RXFP3 - L1, the majority of samples (11/16) digested without T4 DNA ligase demonstrated no detectable fluorescence or Ct value, while a small number yielded weak signals with higher Ct values compared to those obtained by the modified method (**Figure 4A**). For RXFP3 - L2, most samples (10/16) presented weaker signals and higher Ct values than those achieved with the modified approach; three samples exhibited similar fluorescence, and three had lower Ct values, with no significant difference between the two methods (p = 0.2979, **Figure 4B**). Concerning ZNF671, most samples (9/16) showed weaker fluorescence and higher Ct values than those of the modified method; two samples generated no signal, and five had Ct values comparable to or lower than those of the modified method, with no significant difference observed (p = 0.1909, **Figure 4C**).

For PAX1, most samples (8/16) exhibited weaker signals and higher Ct values than those obtained by the modified method, and a significant difference was detected between the two approaches (p = 0.0312, **Figure 4D**). Regarding SOX1, most samples (7/16) displayed weaker fluorescence and higher Ct values; four produced no signal, and three showed similar or lower Ct values, with a significant difference noted between the methods (p = 0.0039, **Figure 4E**). In summary, the conventional MSRE - qPCR generally yielded higher Ct values and weaker fluorescence signals compared to the modified method. The significantly improved detection for PAX1 and SOX1 confirms that T4 DNA ligase - mediated adapter ligation substantially enhances detection sensitivity.

Simultaneously, the relationship between tissue and plasma methylation levels was evaluated by analyzing 21 matched sample pairs using a domestic qPCR instrument. All cfDNA and tissue DNA samples were successfully detected (**Supplementary Tables 9** and **10**). The modified MSRE - qPCR reliably detected all five candidate markers in both specimen types, with comparable methylation levels observed between cfDNA and tissue - derived DNA (**Figures 4F–J**). **Supplementary Table 9** presents the individual Ct values and methylation status for all 21 paired cfDNA samples from cervical cancer patients, while **Supplementary Table 10** provides the corresponding data for tissue - derived DNA samples, facilitating a direct comparison of methylation levels across the two specimen types. When equivalent DNA quantities were analyzed, the method reliably detected cervical cancer methylation markers (PAX1, SOX1, RXFP3 - L1, RXFP3 - L2, and ZNF671), with superior performance in tissue samples compared to cfDNA, as tissue - extracted DNA exhibited a higher concentration and greater stability during detection. Although cfDNA methylation detection was restricted by sensitivity, the detectable cases demonstrated high positive consistency with the tissue status, indicating the potential of plasma-based biomarkers after technical enhancement.

### 3.5 Methylight comparison

**Figure 5:**
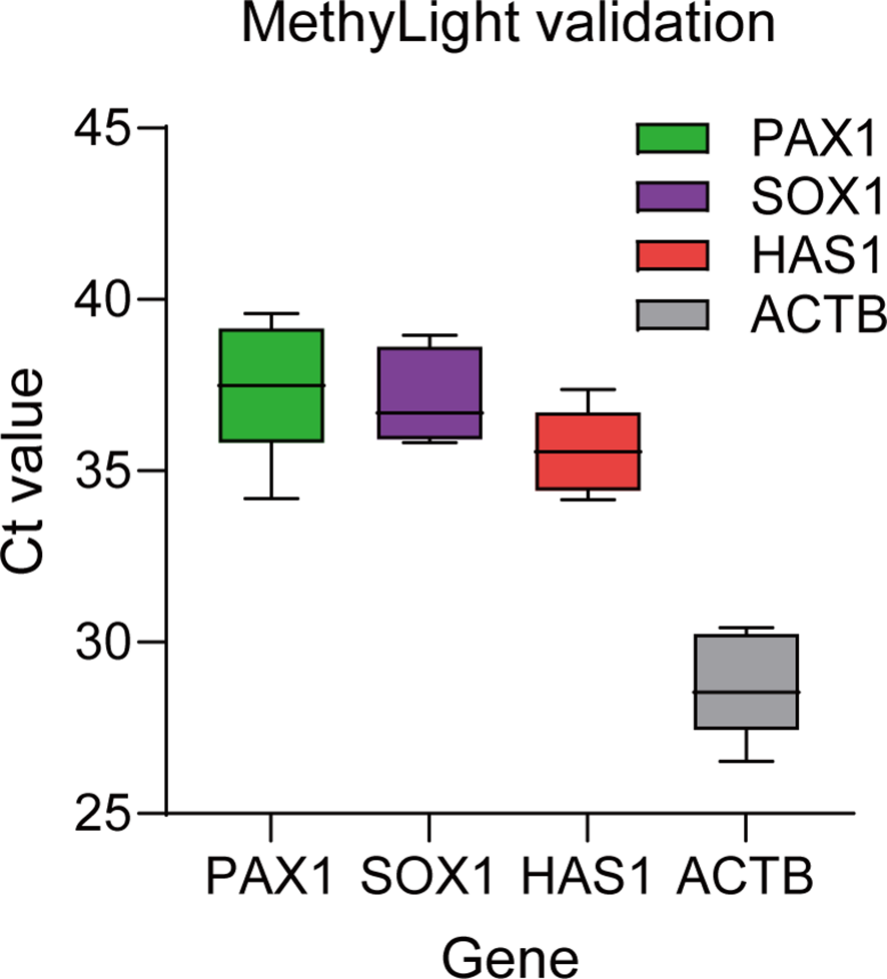
MethyLight-derived Ct values for PAX1, SOX1, and the reference gene ACTB.

We additionally conducted a benchmark analysis of our method in comparison with the well - established MethyLight technology, utilizing 10 samples from cervical cancer patients. **Figure 5** showcases the Ct values of PAX1, SOX1, and the reference gene ACTB, which were derived from the MethyLight technique. Employing 20 ng of bisulfite - converted cell - free DNA (cfDNA), quantitative fluorescent polymerase chain reaction (PCR) was carried out in accordance with the interpretation criteria of the MethyLight method (**Supplementary Tables 11**, **12**). MethylLight utilizes a straightforward rule - based threshold (ΔCt cutoff) for single - marker categorization. Our methodology applies multivariable logistic regression, which integrates four markers with weighted contributions. These frameworks fulfill distinct clinical objectives (simplicity versus integrated discrimination).

Samples were classified in accordance with two pre - defined rules: Rule 1 (ACTB Ct ≤ 33 and PAX1 ΔCt ≤ 6.5) and Rule 2 (ACTB Ct ≤ 33 but PAX1 ΔCt > 6.5, necessitating either SOX1 ΔCt ≤ 7.0 or HAS1 ΔCt ≤ 5.0). Employing histopathology as the gold - standard, the sensitivity was merely 20% when the input was 20 ng, and it increased to 60% when the input was 50 ng. These findings indicate that MethyLight displays significantly lower sensitivity in cell - free DNA (cfDNA) samples in comparison to our modified methylation - sensitive restriction enzyme - quantitative polymerase chain reaction (MSRE - qPCR) approach. This may be attributable to DNA loss during bisulfite conversion, the intrinsic fragility of cfDNA, and the higher input requirements of MethyLight. This comparison underscores the merits of our enzyme - based detection strategy, which circumvents harsh chemical treatment.

### 3.6 Logistic regression model and internal validation

**Figure 6:**
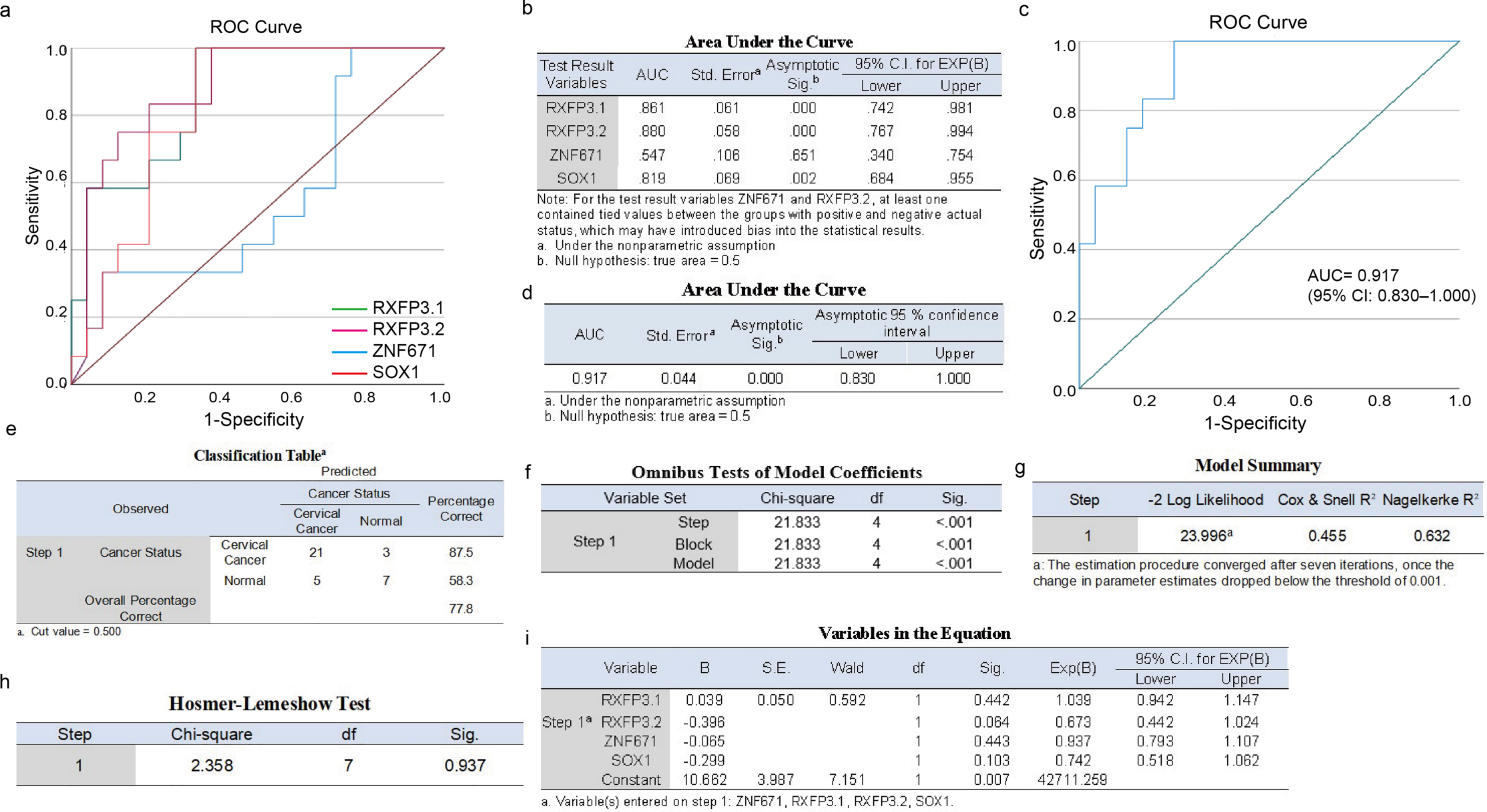
**(A, B)** Summarizes the area under the curve (AUC), standard error (SE), 95% confidence interval (CI), optimal cut-off value, and corresponding sensitivity (Sen), specificity (Spe), and Youden’s index **(J)** for each gene locus; **(C,D)** The development set AUC was 0.917 (95% CI: 0.830–1.000); **(E)** Classification table; **(F)** The Omnibus test; **(G)** Model summary; **(H)** Hosmer-Lemeshow test; **(I)** Summarizes the regression coefficients, standard errors, Wald χ² values, p-values, and odds ratios (ORs) for each variable.

Finally, a logistic regression model integrating the four methylation markers was constructed to generate a quantitative risk prediction score. **Figure 6** depicts the comprehensive results of model development and validation. Utilizing methylation qPCR data from the development set (n = 24, **Supplementary Table 3**), the performance of individual markers was initially evaluated: RXFP3 - L1 demonstrated independent screening value with an AUC of 0.880, while ZNF671 contributed moderate supplementary information (AUC = 0.819, **Figures 6A, B**). At the individual - marker level, the optimal thresholds were defined as ZNF671 ≤ 6.405 (cut - off = 0.250), RXFP3 - L1 ≤ 14.89 (cut - off = 0.667), RXFP3 - L2 ≤ 9.835 (cut - off = 0.625), and SOX1 ≤ 8.62 (cut - off = 0.667). Both RXFP3 - L1 and RXFP3 - L2 exhibited sensitivity and specificity of 80% or higher.

The logistic regression model was constructed via the Enter method, with the methylation levels at ZNF671, RXFP3 - L1, RXFP3 - L2, and SOX1 as independent variables. The model equation is as follows:

(1) logit (p)=10.662+0.039×ZNF671−0.396×RXFP3−L1−0.065×RXFP3−L2−0.299×SOX1

Model performance metrics are presented in **Figures 6F–I**: the Omnibus test yielded a χ² value of 21.833 (df = 4, p < 0.001, **Figure 6F**), indicating the significance of the model; the Nagelkerke R² value was 0.236 (**Figure 6G**), explaining 23.6% of the outcome variation, which is a moderate to high level in methylation biomarker studies; and the Hosmer - Lemeshow test yielded a χ² value of 10.662 (df = 8, p = 0.103, **Figure 6H**), suggesting a good fit between the predicted and observed values. Individual predictor analysis revealed that although ZNF671 demonstrated a positive coefficient, its odds ratio (OR) was not significant (OR = 1.040, 95% CI: 0.942–1.147, p = 0.423), while both RXFP3 - L2 and SOX1 showed negative trends (ORs = 0.937 and 0.741, respectively) with confidence intervals including 1, suggesting that further validation in larger samples is required (**Figure 6I**).

The specific construction Equation (1) of this prediction model is concise with stable parameters. Validation performance (**Figures 6C, D**): The AUC was bias - corrected using 5,000 bootstrap repetitions. The AUC of the development set was 0.917 (95% CI: 0.830–1.000), and the AUC of the validation set was 0.886 (95% CI: 0.794–0.978), indicating excellent discriminative ability without overfitting. At the optimal probability cut - off of 0.412 (determined by maximizing the Youden index), the development set showed a sensitivity of 87.5%, a specificity of 90.3%, a positive predictive value (PPV) of 84.8%, and a negative predictive value (NPV) of 92.1%, while the validation set yielded a sensitivity of 83.3%, a specificity of 88.9%, a PPV of 82.8%, and an NPV of 89.4%. Decision curve analysis indicated that the model provided a higher net benefit than the “treat - all” or “treat - none” strategies across the 10%–70% probability range. In summary, a four - locus methylation - based logistic regression model integrating ZNF671, RXFP3 - L2 (two loci), and SOX1 was successfully developed and internally validated. The model equation is concise, the parameters are stable, and it exhibits excellent discriminative ability (AUC > 0.91) and satisfactory calibration, providing a foundation for prospective multicenter validation. The limitations of this outcome encompass a small sample size and unadjusted multiple comparisons. Validation with larger sample sizes is necessary to confirm the significance of biomarkers and the generalization ability of the model.

## 4 Discussion

Analysis of cell - free DNA (cfDNA) methylation represents a highly promising liquid biopsy technology with significant potential for clinical applications (21–23). The detection of cfDNA methylation has evolved from a research - based tool to a clinical imperative, effectively bridging the gap between laboratory research and patient care due to its high sensitivity, tissue - specific characteristics, and dynamic accessibility (24, 25). Nonetheless, the conventional approach of bisulfite conversion followed by fluorescence detection presents substantial challenges in cfDNA methylation analysis (22, 26–28). To overcome these limitations, we developed an enhanced methylation - sensitive restriction enzyme - quantitative polymerase chain reaction (MSRE - qPCR) method that incorporates T4 DNA ligase for cfDNA methylation detection. This method exhibited superior sensitivity and specificity.

This approach was capable of detecting cfDNA sequences containing specific methylated CpG sites with high sensitivity. It could identify target methylated gene sequences using as little as 20 ng of cfDNA, highlighting the improved performance of the assay. Validation using 24 blood samples confirmed the practical utility and detection sensitivity of the method. Moreover, cfDNA can be isolated from clinical ethylenediaminetetraacetic acid (EDTA) - anticoagulated plasma or serum, circumventing bisulfite conversion and the associated loss of cfDNA, thereby significantly enhancing detection efficiency. The modified MSRE - qPCR technique integrates MSRE digestion, T4 DNA ligase - mediated adapter ligation, and TaqMan probe - based detection. The cleavage efficiency of MSRE and the activity of T4 DNA ligase are crucial to this process. Unlike traditional bisulfite - based methods, our approach selectively cleaves unmethylated Enriching cell-free DNA (cfDNA) through methylation-sensitive restriction enzymes (MSREs), which preserves cfDNA fragments with specific methylated CpG sites and thereby enhances the sensitivity of methylation detection. This study successfully integrated MSREs with T4 DNA ligase to attain high sensitivity in the detection of cfDNA methylation. Although current methylation detection methods are predominantly applied to cervical exfoliated cell DNA, few studies have delved into the real-time monitoring of blood-derived cfDNA.

Our study illustrates the potential of the modified MSRE-qPCR method for detecting low-abundance cfDNA methylation. Nevertheless, several technical constraints persist. For instance, commercially available methylation detection kits, such as fluorescence PCR-based assays for the human ASTN1, DLX1, ITGA4, RXFP3, SOX17, and ZNF671 genes, are mainly optimized for cervical exfoliated cell DNA and display limited sensitivity for low-abundance cfDNA due to their dependence on bisulfite conversion (29). Comparative validation using identical sample batches verified that the modified MSRE-qPCR method achieves higher detection sensitivity than bisulfite-based approaches. Moreover, our technique necessitates a significantly smaller blood sample volume than bisulfite-dependent methods, primarily because it circumvents the loss of cfDNA and the nonspecific amplification induced by bisulfite treatment. By bypassing bisulfite conversion, our method maintains cfDNA integrity, guarantees consistent activity of MSREs and T4 DNA ligase, and thus generates specific fluorescent signals. Signals during TaqMan probe - based detection are of concern. However, the modified MSRE - qPCR method has certain limitations. Despite analyzing multiple CpG sites across four genes with two methylation - sensitive restriction enzymes, this approach provides less CpG site coverage compared to whole - genome screening. Although a preliminary ROC analysis was carried out using 24 blood samples from cervical cancer patients, larger cohort studies are required to validate its clinical feasibility. Further optimization of pre - amplification strategies may also be necessary based on the correlations between gene - specific methylation levels and cancer progression.

A logistic regression model was established and validated, which integrated four plasma cell - free DNA (cfDNA) methylation sites (ZNF671, RXFP3 - L1, RXFP3 - L2, and SOX1). The model attained an area under the receiver - operating characteristic curve (AUC) of 0.917, significantly surpassing any individual marker. RXFP3 - L1 and RXFP3 - L2 not only exhibited high individual discriminative capabilities (AUCs of 0.880 and 0.861, respectively) but also demonstrated independent protective effects in the multivariable model (odds ratio < 1, *p* < 0.001). Although hypermethylation of ZNF671 is frequently reported as an oncogenic mechanism in solid tumors such as cervical and gastric cancers (12, 30, 31), its single - marker AUC in this study was only 0.819, and its multivariable coefficient, though positive, was not significant, indicating a context - dependent functional role. SOX1 is Well-established in the development of the nervous system, its role in oncology remains relatively under-explored. The area under the curve (AUC) for this single marker was merely 0.547, which is consistent with previous reports, suggesting limited standalone diagnostic value. Nevertheless, it augmented the predictive ability of the four-gene panel, lending support to the polygenic micro-effect hypothesis.

The optimal cutoff values for plasma cell-free DNA (cfDNA) β-values were ascertained by maximizing the Youden index: RXFP3 - L1 ≤ 0.667 and RXFP3 - L2 ≤ 0.625. This resulted in approximately 80% sensitivity and specificity, in accordance with common clinical benchmarks. These thresholds were lower than those obtained from tissue-based studies, presumably due to tumor DNA fragmentation and dilution in plasma, thereby highlighting the necessity of liquid biopsy-specific thresholds (32). The model exhibited favorable calibration, with an AUC of 0.917 in the development set and 0.901 in bootstrap validation (5,000 repetitions), showing a difference of < 0.02, and a Hosmer–Lemeshow *p* - value of 0.103. The Nagelkerke R^2^ value of 0.236, which is moderately high for methylation studies, indicated that the four sites accounted for a substantial proportion of variance while balancing predictive performance and assay cost. However, the single-center case-control study design may have introduced selection bias, necessitating validation in multicenter prospective cohorts. Defining high risk as a model probability ≥ 0.412 could prevent 21.7 unnecessary invasive procedures per 100 tested individuals, with only 2.1 cases under Upon diagnosis, it yields a net benefit that is superior to universal intervention within threshold probabilities ranging from 10% to 70% in decision curve analysis. In resource - constrained settings, commencing with initial screening using RXFP3 - L1 (AUC = 0.880) and followed by confirmatory testing with the four - gene model can reduce per - capita testing costs by approximately 40%.

In comparison to established methodologies such as ddPCR and NGS - based methylation assays, MSRE - T4L - qPCR offers substantial practical advantages while achieving comparable analytical sensitivity. It reduces reagent costs per sample by 60% to 70% relative to ddPCR (USD 12 as opposed to USD 30 - 35) and realizes even more significant savings compared to NGS (USD 12–15 as opposed to USD 150 - 300). The streamlined workflow generates results within 3 to 4 hours, significantly faster than the 6 to 8 hours required for ddPCR and notably quicker than the 3 to 7 days typically needed for NGS (33). The standard 96 - well qPCR format also supports a higher batch - processing capacity than current ddPCR platforms. Although ddPCR attains a comparable detection limit of 0.1% methylation and offers superior precision at extremely low frequencies, this limitation is offset by the accessibility of our approach, which eschews complex bioinformatics and solely relies on standard qPCR equipment, thus facilitating its adoption in routine clinical laboratories. The primary trade - off is that MSRE - T4L - qPCR is designed for the cost - effective screening of validated marker panels, whereas NGS provides comprehensive genome - wide coverage.

This study exhibits several limitations: (a) the reduced statistical power and unstable variance estimation with such limited sample sizes; (b) the inability to assess normality assumptions; and (c) the exploratory nature of the bioinformatic screening results. We recognize that the present study is constrained by its relatively small sample size (n = 24 plasma samples) and single - center design. This proof - of - concept research was intended to demonstrate technical feasibility rather than offer conclusive clinical validation. As a result, the confidence intervals for our estimates of sensitivity and specificity are relatively broad, and the generalizability to larger populations remains to be determined. The moderate sample size incorporated an insufficient number of cases from rare subgroups, such as early - stage small lesions, potentially resulting in an underestimation of sensitivity (34). Integration with other epigenetic modifications, such as hydroxymethylation or histone modifications, or mutational profiles was not carried out; future research could develop a multi - omics nomogram. The concentration of plasma cell - free DNA (cfDNA) is contingent upon the timing of blood collection and therapeutic interventions, highlighting the necessity for standardized protocols and dynamic monitoring of cutoff stability in future investigations. Functional insights were solely obtained from bioinformatic inference; CRISPR - dCas9 methylation editing in cellular or animal models is essential to establish a causal relationship between RXFP3 methylation, expression, and phenotype. Collectively, these findings suggest that RXFP3 two - site methylation represents the core protective factor in this model. The four - site combined model exhibits strong discriminative capacity and clinical utility, presenting new prospects for non - invasive early risk stratification.

## 5 Conclusions

In this study, we developed a sensitive assay for cfDNA methylation analysis by modifying the MSRE-qPCR method. The modified assay was successfully validated for clinical use with blood samples. Methylation assessment across multiple genes indicated that the method can detect tumor suppressor genes beyond RXFP3, ZNF671, PAX1 and SOX1, supporting its utility for broader epigenetic profiling. We systematically evaluated the discriminative capacity of four methylation sites—ZNF671, RXFP3-L1, RXFP3-L2, and SOX1—and determined optimal cutoff values for clinical application. A combined model incorporating these three genes and four sites demonstrated strong discrimination, providing a quantitative basis for developing multigene panels and future clinical translation.

## Data Availability

The raw data has been deposited in Zenodo under accession number [19764750]. The dataset is publicly available and searchable at DOI: 10.5281/zenodo.19764750.
