# Supplementary material for: Application of a modified MSRE-qPCR method for detecting circulating cell-free DNA methylation in cervical cancer
Source: Front Oncol. 2026 May 4;16:1759488. doi: 10.3389/fonc.2026.1759488 (PMC13180608; doi:10.3389/fonc.2026.1759488)
Supplement: Supplementary file 1 [file DataSheet1.docx]

***Supplemental Figures***

***
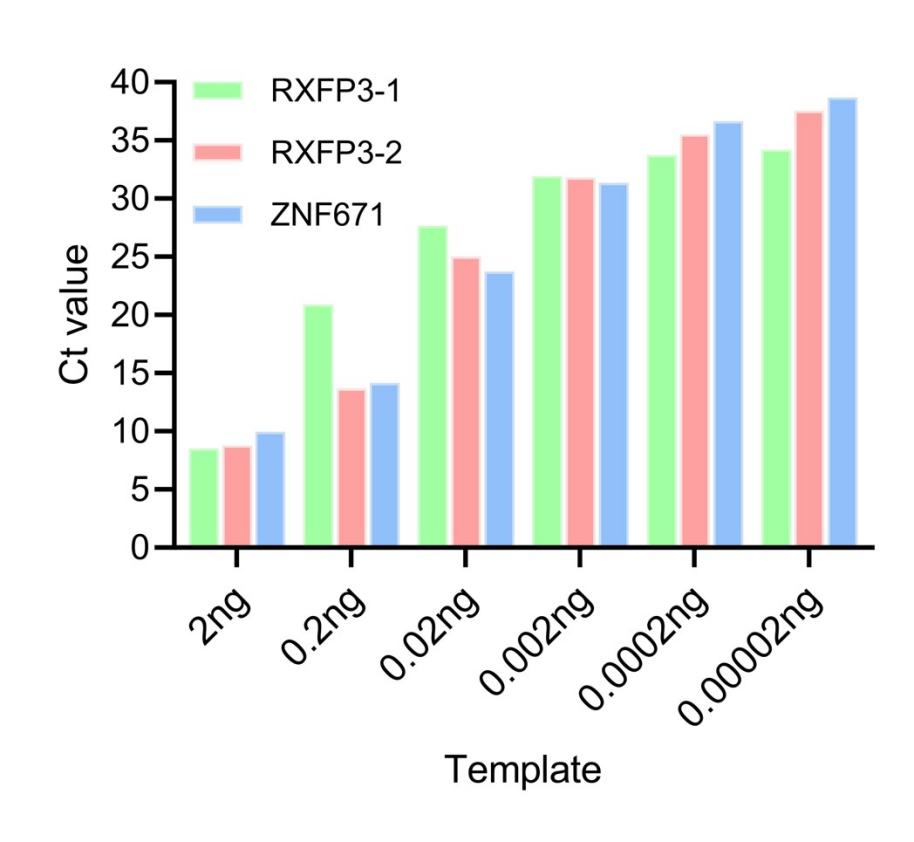
***

**Figure s1a:** The detection limit of modified MSRE-qPCR was improved following the gradient dilution of three cfDNA gene templates.

***
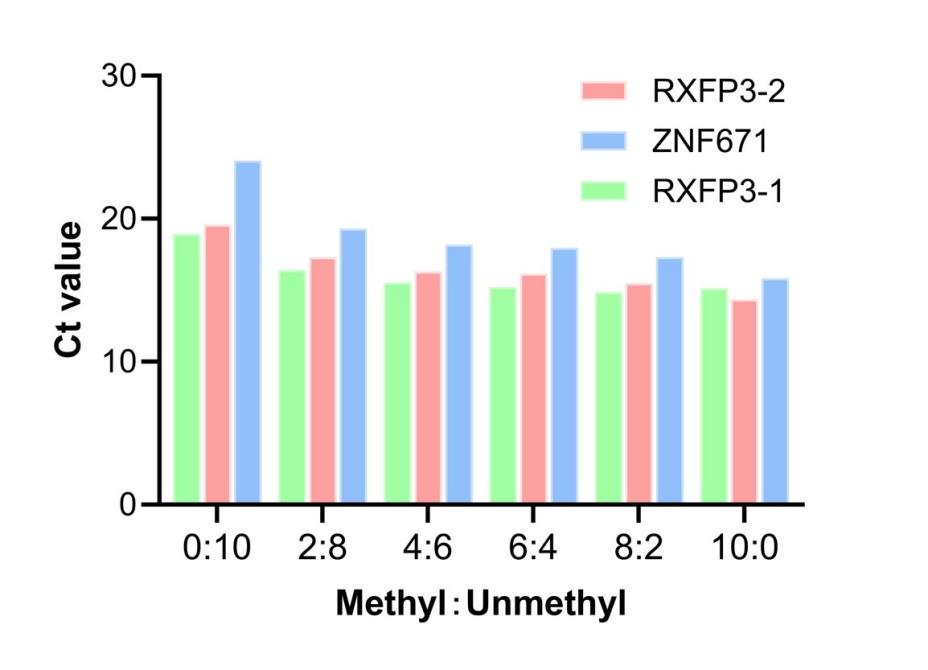
***

**Figure s1b:** The modified MSRE-qPCR method was refined to detect the cycle threshold (Ct) values for each gene across mixtures of methylated and unmethylated DNA.

***
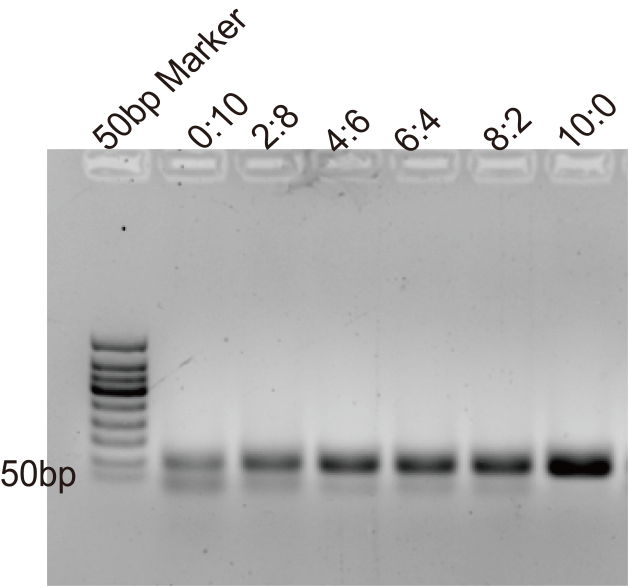
***

**Figure s2a:** Proportional amplification was validated by Sepharose gel electrophoresis (Figure S1b). The label "0:10" denotes a DNA sample containing 0 parts methylated and 10 parts unmethylated DNA. "2:8" represents a mixture of 2 parts methylated and 8 parts unmethylated DNA. The "4:6" sample contains 4 parts methylated and 6 parts unmethylated DNA. "6:4" corresponds to 6 parts methylated and 4 parts unmethylated DNA. "8:2" refers to 8 parts methylated and 2 parts unmethylated DNA. Finally, "10:0" indicates a mixture of 10 parts methylated and 0 parts unmethylated DNA.

***
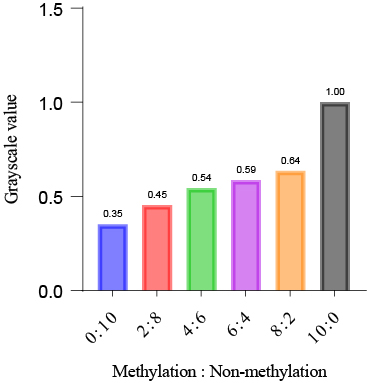
***

**Figure s2b:** ImageJ was used to verify DNA amplification by performing a grayscale evaluation of Figure S2a.

***
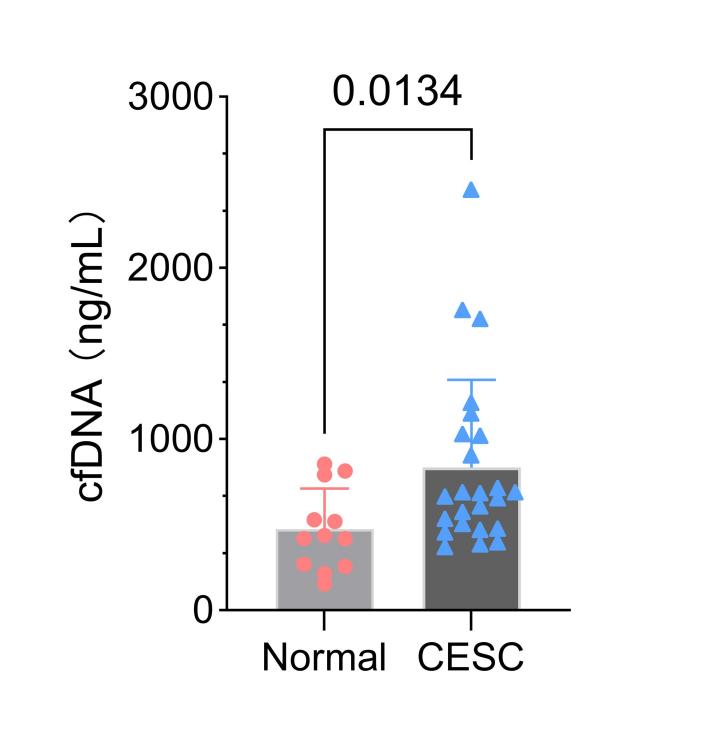
***

**Figure s3:** A comparison of cfDNA concentrations between the NC and CC groups was conducted.
