# Supplementary material for: Application of a modified MSRE-qPCR method for detecting circulating cell-free DNA methylation in cervical cancer
Source: Front Oncol. 2026 May 4;16:1759488. doi: 10.3389/fonc.2026.1759488 (PMC13180608; doi:10.3389/fonc.2026.1759488)
Supplement: Supplementary file 2 [file DataSheet2.docx]

***Supplemental Tables***

| Table S1 : Detailed data(DNMIVD) for four Methylation Gene of Cervical Cancer | | | | |
| --- | --- | --- | --- | --- |
| Disease | Gene | difference | adjusted—pvalue | DM-type |
| CESC | RXFP3 | 0.483121 | 0.0005394 | Hypermethylation |
| CESC | ZNF671 | 0.342626 | 0.0776273 | Not Sig |
| CESC | PAX1 | 0.494863 | 4.139e-7 | Hypermethylation |
| CESC | SOX1 | 0.41701 | 4.507e-7 | Hypermethylation |
| Note：Significance was determined by Fisher's exact test comparing cancer vs. normal. | | | | |

| Table S2 : Detailed data for nine CpG sites | | | | | | | | |
| --- | --- | --- | --- | --- | --- | --- | --- | --- |
| CpG | Chromosome | Location | Relation  to CpG island | Genomic Group | Gene Symbol | average  beta value  (CESC) | average  beta value  Healthy) | adjusted P-value |
| cg26390889 | 5 | 33936462 | Island | TSS200 | RXFP3 | 0.548835 | 0.048333 | 7.68E-05 |
| cg26986911 | 5 | 33936292 | Island | TSS200 |  | 0.464385 | 0.015333 | 3.11E-02 |
| cg07832473 | 5 | 33936253 | Island | TSS200 |  | 0.487537 | 0.019666 | 1.06E-02 |
| cg15408073 | 5 | 33936322 | Island | TSS200 |  | 0.484116 | 0.051666 | 2.15E-04 |
| cg11977686 | 19 | 58238987 | Island | 1stExon;5'UTR | ZNF671 | 0.516940 | 0.033492 | 6.43E-03 |
| cg19246110 | 19 | 58238928 | Island | 1stExon;5'UTR |  | 0.597755 | 0.018864 | 3.09E-04 |
| cg22906273 | 20 | 21686253 | Island | TSS200 | PAX1 | 0.381579 | 0.020666 | 7.46E-03 |
| cg21385666 | 13 | 112724270 | Island | 3'UTR;1stExon | SOX1 | 0.463381 | 0.050666 | 4.71E-03 |
| cg23668285 | 13 | 112724245 | Island | 3'UTR;1stExon |  | 0.515061 | 0.236333 | 3.49E-03 |
| Reference Gene : UCSC GRCh37/hg19 | | | | | | | | |

| Table S3 : Detailed information of Cervical Cancer patients and Controls | | | |
| --- | --- | --- | --- |
| No. | Sample number | Pathological diagnosis | cfDNA concentration （ng/mL） |
| CC-01 | ZL20140001300/ZL20140001304 | Papillary squamous cell carcinoma of the cervix（Ib2） | 473.33 |
| CC-02 | ZL20140006095/ZL20140006094 | Moderately differentiated squamous cell carcinoma of the cervix（Ib1） | 392.85 |
| CC-03 | ZL20140006026 | Moderately differentiated squamous cell carcinoma of the cervix（IIa1） | 1207.50 |
| CC-04 | ZL20140006058 | Cervical squamous cell carcinoma of low to intermediate differentiation（Ib2） | 686.87 |
| CC-05 | ZL20140006077/ZL20140006081 | Moderately differentiated squamous cell carcinoma of the cervix（Ib1） | 1026.66 |
| CC-06 | ZL20140006017/ZL20140006089 | Cervical squamous cell carcinoma of intermediate to low differentiation（Ib2） | 2453.50 |
| CC-07 | ZL20140005864/ZL20140005905 | Highly differentiated adenocarcinoma of the cervical canal（IIIc1） | 1146.25 |
| CC-08 | ZL20140005918 | Cervical poorly differentiated squamous cell carcinoma | 710 |
| CC-09 | ZL20140005801 | Cervical malignant tumor | 686 |
| CC-10 | ZL20140005859 | Moderately differentiated adenocarcinoma of the cervix（Ib1） | 660 |
| CC-11 | ZL20140005781 | Cervical poorly differentiated squamous cell carcinoma | 570 |
| CC-12 | ZL20140005719 | High-to-moderately differentiated squamous cell carcinoma of the cervix（Ib2） | 380 |
| CC-13 | ZL20140005721 | High-to-moderately differentiated squamous cell carcinoma of the cervix（IIa1） | 530 |
| CC-14 | ZL20140005681 | Moderately differentiated adenocarcinoma of the cervix（Ib2） | 650 |
| CC-15 | ZL20140005709 | Moderately differentiated squamous cell carcinoma of the cervix | 450 |
| CC-16 | ZL20140005837 | Cervical poorly differentiated squamous cell carcinoma（Ib1） | 1750 |
| CC-17 | ZL20140005544/ZL20140005557 | Cervical malignant tumor | 680 |
| CC-18 | ZL20140005576 | Moderately differentiated squamous cell carcinoma of the cervix（Ib1） | 365.55 |
| CC-19 | ZL20140005525 | Serous papillary adenocarcinoma of the cervical canal（IIa） | 500 |
| CC-20 | ZL20140005941 | Cervical malignant tumor | 1697.50 |
| CC-21 | ZL20140005531 | Cervical poorly differentiated squamous cell carcinoma（IIa1） | 466.66 |
| CC-22 | ZL20140005096/ZL20140005168 | Moderately differentiated squamous cell carcinoma of the cervix（Ib1） | 603.75 |
| CC-23 | ZL20140005139 | Cervical malignant tumor | 900 |
| CC-24 | ZL20140005588 | Cervical poorly differentiated squamous cell carcinoma（Ib1） | 1015 |
| NC-01 | 25062150875 | Healthy | 214.28 |
| NC-02 | 25062150875 | Healthy | 528.57 |
| NC-03 | 25062150827 | Healthy | 257.14 |
| NC-04 | 25052854934 | Healthy | 519.04 |
| NC-05 | 25062353509 | Healthy | 154.54 |
| NC-06 | 25062450533 | Healthy | 854.54 |
| NC-07 | 25062655005 | Healthy | 418.18 |
| NC-08 | 25062650329 | Healthy | 268 |
| NC-09 | 32505210844 | Healthy | 419.04 |
| NC-10 | 32506211170 | Healthy | 435 |
| NC-11 | 25062652422 | Healthy | 813.63 |
| NC-12 | 25062651214 | Healthy | 790.90 |
| Note: Extract cfDNA from 600 μL - 1.5 mL of blood and elute it with 70 - 100 μL of DEPC H₂O. | | | |

| Table S4 : Detailed information of Cervical Cancer patients tissue samples | | | |
| --- | --- | --- | --- |
| No. | Sample number | Pathological diagnosis | tiDNA concentration （ng/μL） |
| CC-01 | ZL20140001258 | papillary squamous cell carcinoma of the cervix(Ib2) | 22.00 |
| CC-02 | - | Moderately differentiated squamous cell carcinoma of the cervix(Ib1) | - |
| CC-03 | ZL20140001007 | Moderately differentiated squamous cell carcinoma of the cervix(IIa1) | 8.80 |
| CC-04 | - | Cervical squamous cell carcinoma of low to intermediate differentiation(Ib2) | - |
| CC-05 | ZL20140001190 | Moderately differentiated squamous cell carcinoma of the cervix(Ib1) | 12.10 |
| CC-06 | ZL20140001110 | Cervical squamous cell carcinoma of intermediate to low differentiation(Ib2) | 10.30 |
| CC-07 | ZL20140001034 | Highly differentiated adenocarcinoma of the cervical canal(IIIc1) | 13.40 |
| CC-08 | ZL20140000998 | Cervical poorly differentiated squamous cell carcinoma | 19.60 |
| CC-09 | ZL20140000918 | Cervical malignant tumor | 10.10 |
| CC-10 | ZL20140000913 | Moderately differentiated adenocarcinoma of the cervix(Ib1) | 22.60 |
| CC-11 | ZL20140000823 | Cervical poorly differentiated squamous cell carcinoma | 15.30 |
| CC-12 | ZL20140000676 | High-to-moderately differentiated squamous cell carcinoma of the cervix(Ib2) | 9.40 |
| CC-13 | ZL20140000777 | High-to-moderately differentiated squamous cell carcinoma of the cervix(IIa1) | 13.80 |
| CC-14 | ZL20140000568 | Moderately differentiated adenocarcinoma of the cervix(Ib2) | 7.80 |
| CC-15 | ZL20140000709 | Moderately differentiated squamous cell carcinoma of the cervix | 6.40 |
| CC-16 | ZL20140000845 | Cervical poorly differentiated squamous cell carcinoma(Ib1) | 6.60 |
| CC-17 | ZL20140000466 | Cervical malignant tumor | 21.20 |
| CC-18 | ZL20140000474 | Moderately differentiated squamous cell carcinoma of the cervix(Ib1) | 24.30 |
| CC-19 | ZL20140000344 | Serous papillary adenocarcinoma of the cervical canal(IIa) | 13.50 |
| CC-20 | - | Cervical malignant tumor | - |
| CC-21 | ZL20140000461 | Cervical poorly differentiated squamous cell carcinoma(IIa1) | 21.30 |
| CC-22 | ZL20140000189 | Moderately differentiated squamous cell carcinoma of the cervix(Ib1) | 16.80 |
| CC-23 | ZL20140000179 | Cervical malignant tumor | 14.50 |
| CC-24 | ZL20140000396 | Cervical poorly differentiated squamous cell carcinoma(Ib1) | 8.90 |
| Note: 20mg of cervical lesion tissue sample was extracted using 200μL DEPC water elution. | | | |

| Table S5: The sequences of the primers and probes for the selected nine sites and for internal controls | | | | |
| --- | --- | --- | --- | --- |
| No. | Target site | Forward primer | Reverse primer | Probe |
| 1 | chr5: 33936462 | CCT CAT CCA AGC AGT CCC | GAA TGC GAT CTT GCG CTC | GGG GCC GCT CGC TCC C |
| 2 | Chr5: 33936292 | GCG ATC TTG CGC CGC CTT G | CGT CTC TCC GCG GTT GTC | GCC AGC GGC TCT CAC C |
| 3 | Chr5: 33936253 |  |  |  |
| 4 | Chr5: 33936322 |  |  |  |
| 5 | Chr19:58238987 | GAA TGC GAT CTT GCG CGC TTT C | GTA GCG GAC ATT TTG TTT CTG T | GTG GGC CGC AGG T |
| 6 | chr19:58238928 |  |  |  |
| 7 | Chr20:21686253 | CCA AAG GGC CGC AGT GAC | GAC GTG TCC TCC ACG TCA ATC TC | CAC GCC GGA GAC GCG C |
| 8 | Chr13:112724270 | GGC TCT GAC GTT ACC TTG C | CTT CCT CCT CCC TCC TCT GG | CAG GTG GAA GGC GCC CCG C |
| 9 | Chr13:112724245 |  |  |  |
| Control |  | CCA CTC CTC CAC CTT TGA CG | CTG GTG GTC CAG GGG TCT TA | CCA TGA GGT CCA CCA CCC |
| chr : chromosome, Reference Gene : UCSC GRCh37/hg19 | | | | |

| Table S6: Results of the evaluation of primers and probes specificity for Improved MSRE - qPCR detection ((ABI 7500) | | | | | | | |
| --- | --- | --- | --- | --- | --- | --- | --- |
|  | | First time | | Second time | | Third time | |
| Gene | Sample Name | Instrument/ABI7500 | Target Ct | Instrument/ABI7500 | Target Ct | Instrument/ABI7500 | Target Ct |
| PAX1 | Sample |  |  |  |  |  |  |
|  | Sample1 | 1 | 14.92 | 1 | 16.32 | 1 | 16.31 |
|  | Sample2 | 1 | 17.84 | 1 | 17.88 | 1 | 18.35 |
|  | Sample3 | 1 | 17.68 | 1 | 18.01 | 1 | 18.10 |
|  | Sample4 | 1 | 16.79 | 1 | 16.91 | 1 | 12.52 |
|  | Sample5 | 1 | 17.67 | 1 | 17.07 | 1 | 17.42 |
|  | Sample6 | 1 | 17.35 | 1 | 16.63 | 1 | 17.26 |
|  | Sample7 | 1 | 16.51 | 1 | 16.23 | 1 | 16.22 |
|  | Sample8 | 1 | 16.55 | 1 | 16.36 | 1 | 16.43 |
|  | Sample9 | 1 | 17.84 | 1 | 18.06 | 1 | 17.59 |
|  | Sample10 | 1 | 17.51 | 1 | 17.41 | 1 | 17.57 |
|  | Sample11 | 1 | 17.08 | 1 | 17.02 | 1 | 17.03 |
|  | Sample12 | 1 | 17.79 | 1 | 17.55 | 1 | 15.64 |
|  | Sample13 | 1 | 18.42 | 1 | 18.28 | 1 | 18.30 |
|  | Sample14 | 1 | 17.08 | 1 | 16.59 | 1 | 16.75 |
|  | Sample15 | 1 | 16.69 | 1 | 16.75 | 1 | 14.76 |
|  | Sample16 | 1 | 19.13 | 1 | 18.35 | 1 | 17.97 |
| SOX1 | Sample |  |  |  |  |  |  |
|  | Sample1 | 1 | 4.64 | 1 | 4.65 | 1 | 4.57 |
|  | Sample2 | 1 | 4.65 | 1 | 4.57 | 1 | 4.59 |
|  | Sample3 | 1 | 4.60 | 1 | 4.61 | 1 | 4.63 |
|  | Sample4 | 1 | 4.64 | 1 | 4.61 | 1 | 4.62 |
|  | Sample5 | 1 | 4.74 | 1 | 4.94 | 1 | 4.77 |
|  | Sample6 | 1 | 4.95 | 1 | 5.34 | 1 | 4.99 |
|  | Sample7 | 1 | 4.70 | 1 | 4.64 | 1 | 4.35 |
|  | Sample8 | 1 | 4.67 | 1 | 4.82 | 1 | 4.74 |
|  | Sample9 | 1 | 5.80 | 1 | 5.64 | 1 | 5.65 |
|  | Sample10 | 1 | 5.27 | 1 | 5.17 | 1 | 5.32 |
|  | Sample11 | 1 | 13.73 | 1 | 13.36 | 1 | 13.40 |
|  | Sample12 | 1 | 13.32 | 1 | 13.38 | 1 | 13.62 |
|  | Sample13 | 1 | 18.25 | 1 | 18.31 | 1 | 18.26 |
|  | Sample14 | 1 | 17.02 | 1 | 17.32 | 1 | 16.75 |
|  | Sample15 | 1 | 16.66 | 1 | 16.31 | 1 | 16.02 |
|  | Sample16 | 1 | 12.34 | 1 | 12.24 | 1 | 12.33 |
|  | Sample17 | 1 | 14.95 | 1 | 16.68 | 1 | 17.42 |
|  | Sample18 | 1 | 13.09 | 1 | 14.92 | 1 | 15.5 |
|  | Sample19 | 1 | 5.57 | 1 | 6.37 | 1 | 6.49 |
|  | Sample20 | 1 | 4.78 | 1 | 5.35 | 1 | 5.33 |
|  | Sample21 | 1 | 4.88 | 1 | 5.44 | 1 | 5.46 |
|  | Sample22 | 1 | 4.67 | 1 | 5.71 | 1 | 6.17 |
|  | Sample23 | 1 | 4.82 | 1 | 5.62 | 1 | 5.69 |
|  | Sample24 | 1 | 4.60 | 1 | 5.19 | 1 | 5.40 |
| ZNF671 | Sample |  |  |  |  |  |  |
|  | Sample1 | 1 | 11.55 | 1 | 11.74 | 1 | 16.00 |
|  | Sample2 | 1 | 13.01 | 1 | 12.94 | 1 | N/A |
|  | Sample3 | 1 | 18.01 | 1 | 18.13 | 1 | 17.67 |
|  | Sample4 | 1 | 17.64 | 1 | 17.51 | 1 | 17.57 |
|  | Sample5 | 1 | 14.05 | 1 | 14.48 | 1 | 14.26 |
|  | Sample6 | 1 | 14.23 | 1 | 14.48 | 1 | 14.50 |
|  | Sample7 | 1 | 14.41 | 1 | 14.80 | 1 | 14.64 |
|  | Sample8 | 1 | 14.44 | 1 | 14.44 | 1 | 14.33 |
|  | Sample9 | 1 | 20.17 | 1 | 20.05 | 1 | 20.26 |
|  | Sample10 | 1 | 19.04 | 1 | 19.47 | 1 | 19.48 |
|  | Sample11 | 1 | 13.37 | 1 | 13.25 | 1 | 13.31 |
|  | Sample12 | 1 | 14.93 | 1 | 15.01 | 1 | 14.85 |
|  | Sample13 | 1 | 14.14 | 1 | 14.07 | 1 | 14.39 |
|  | Sample14 | 1 | 11.28 | 1 | 11.26 | 1 | 11.33 |
|  | Sample15 | 1 | 16.38 | 1 | 16.88 | 1 | 17.04 |
|  | Sample16 | 1 | 13.52 | 1 | 13.72 | 1 | 12.18 |
|  | Sample17 | 1 | N/A | 1 | N/A | 1 | N/A |
|  | Sample18 | 1 | 6.15 | 1 | 6.28 | 1 | 5.99 |
|  | Sample19 | 1 | 12.42 | 1 | 12.43 | 1 | 11.44 |
|  | Sample20 | 1 | 7.90 | 1 | 8.43 | 1 | 8.56 |
|  | Sample21 | 1 | 8.94 | 1 | 9.55 | 1 | 9.72 |
|  | Sample22 | 1 | 9.58 | 1 | 11.09 | 1 | 11.09 |
|  | Sample23 | 1 | 11.58 | 1 | 11.64 | 1 | 10.43 |
|  | Sample24 | 1 | 11.68 | 1 | 11.64 | 1 | 10.23 |
| RXFP3-L1 | Sample |  |  |  |  |  |  |
|  | Sample1 | 1 | 10.98 | 1 | 10.77 | 1 | 10.48 |
|  | Sample2 | 1 | 10.63 | 1 | 10.49 | 1 | 10.3 |
|  | Sample3 | 1 | 10.05 | 1 | 9.86 | 1 | 9.90 |
|  | Sample4 | 1 | 10.39 | 1 | 10.20 | 1 | 10.22 |
|  | Sample5 | 1 | 10.47 | 1 | 10.26 | 1 | 10.61 |
|  | Sample6 | 1 | 10.52 | 1 | 10.52 | 1 | 10.43 |
|  | Sample7 | 1 | 8.55 | 1 | 8.36 | 1 | 8.72 |
|  | Sample8 | 1 | 9.40 | 1 | 9.18 | 1 | 8.64 |
|  | Sample9 | 1 | 15.13 | 1 | 14.56 | 1 | 15.81 |
|  | Sample10 | 1 | 16.41 | 1 | 17.10 | 1 | 16.82 |
|  | Sample11 | 1 | 13.55 | 1 | 13.58 | 1 | 13.43 |
|  | Sample12 | 1 | 20.97 | 1 | 21.66 | 1 | 21.02 |
|  | Sample13 | 1 | 15.77 | 1 | 16.48 | 1 | 15.75 |
|  | Sample14 | 1 | 17.04 | 1 | 16.99 | 1 | 16.90 |
|  | Sample15 | 1 | 16.31 | 1 | 16.33 | 1 | 16.31 |
|  | Sample16 | 1 | 15.32 | 1 | 15.24 | 1 | 15.34 |
|  | Sample17 | 1 | 12.95 | 1 | 13.08 | 1 | 13.20 |
|  | Sample18 | 1 | 12.00 | 1 | 12.06 | 1 | 11.99 |
|  | Sample19 | 1 | 12.12 | 1 | 12.11 | 1 | 12.13 |
|  | Sample20 | 1 | 11.56 | 1 | 11.49 | 1 | 11.58 |
|  | Sample21 | 1 | 12.97 | 1 | 13.02 | 1 | 12.96 |
|  | Sample22 | 1 | 14.75 | 1 | 14.78 | 1 | 14.98 |
|  | Sample23 | 1 | 15.44 | 1 | 15.28 | 1 | 15.38 |
|  | Sample24 | 1 | 14.72 | 1 | 14.87 | 1 | 14.87 |
| RXFP3-L2 | Sample |  |  |  |  |  |  |
|  | Sample1 | 1 | 5.64 | 1 | 5.49 | 1 | 5.45 |
|  | Sample2 | 1 | 5.64 | 1 | 5.50 | 1 | 5.37 |
|  | Sample3 | 1 | 5.16 | 1 | 5.21 | 1 | 5.12 |
|  | Sample4 | 1 | 5.22 | 1 | 5.33 | 1 | 5.40 |
|  | Sample5 | 1 | 6.25 | 1 | 6.21 | 1 | 6.27 |
|  | Sample6 | 1 | 5.58 | 1 | 5.57 | 1 | 5.65 |
|  | Sample7 | 1 | 6.51 | 1 | 6.55 | 1 | 6.55 |
|  | Sample8 | 1 | 8.46 | 1 | 8.54 | 1 | 8.29 |
|  | Sample9 | 1 | 5.53 | 1 | 5.57 | 1 | 5.23 |
|  | Sample10 | 1 | 6.26 | 1 | 6.31 | 1 | 6.32 |
|  | Sample11 | 1 | 11.35 | 1 | 11.35 | 1 | 11.30 |
|  | Sample12 | 1 | 13.01 | 1 | 13.05 | 1 | 13.08 |
|  | Sample13 | 1 | 13.31 | 1 | 13.34 | 1 | 13.36 |
|  | Sample14 | 1 | 11.07 | 1 | 10.99 | 1 | 10.65 |
|  | Sample15 | 1 | 12.96 | 1 | 12.81 | 1 | 12.32 |
|  | Sample16 | 1 | 11.18 | 1 | 11.18 | 1 | 11.03 |
|  | Sample17 | 1 | 12.05 | 1 | 11.85 | 1 | 12.50 |
|  | Sample18 | 1 | 10.87 | 1 | 10.85 | 1 | 10.13 |
|  | Sample19 | 1 | 9.34 | 1 | 9.87 | 1 | 9.31 |
|  | Sample20 | 1 | 7.31 | 1 | 7.71 | 1 | 7.89 |
|  | Sample21 | 1 | N/A | 1 | N/A | 1 | N/A |
|  | Sample22 | 1 | 7.20 | 1 | 7.84 | 1 | 7.82 |
|  | Sample23 | 1 | 6.98 | 1 | 7.12 | 1 | 7.11 |
|  | Sample24 | 1 | 7.08 | 1 | 7.51 | 1 | 7.67 |
| GAPDH | Sample |  |  |  |  |  |  |
|  | Sample1 | 1 | 17.82 | 1 | 17.42 | 1 | 17.33 |
|  | Sample2 | 1 | 21.69 | 1 | 21.56 | 1 | 21.42 |
|  | Sample3 | 1 | 23.03 | 1 | 22.78 | 1 | 22.10 |
|  | Sample4 | 1 | 21.94 | 1 | 22.27 | 1 | 23.16 |
|  | Sample5 | 1 | 22.79 | 1 | 22.57 | 1 | 22.57 |
|  | Sample6 | 1 | 20.52 | 1 | 19.69 | 1 | 19.65 |
|  | Sample7 | 1 | 24.67 | 1 | 24.37 | 1 | 25.29 |
|  | Sample8 | 1 | N/A | 1 | N/A | 1 | N/A |
|  | Sample9 | 1 | 36.50 | 1 | 37.55 | 1 | 37.58 |
|  | Sample10 | 1 | 18.23 | 1 | 18.02 | 1 | 18.22 |
|  | Sample11 | 1 | 19.63 | 1 | 19.19 | 1 | 18.91 |
|  | Sample12 | 1 | 15.10 | 1 | 15.16 | 1 | 15.10 |
|  | Sample13 | 1 | 19.10 | 1 | 18.79 | 1 | 18.64 |
|  | Sample14 | 1 | 19.27 | 1 | 19.26 | 1 | 19.31 |
|  | Sample15 | 1 | 12.22 | 1 | 13.80 | 1 | 13.84 |
|  | Sample16 | 1 | 16.65 | 1 | 16.43 | 1 | 16.26 |
|  | Sample17 | 1 | 19.49 | 1 | 19.55 | 1 | 18.89 |
|  | Sample18 | 1 | 19.25 | 1 | 19.44 | 1 | 19.27 |
|  | Sample19 | 1 | 20.43 | 1 | 20.33 | 1 | 20.32 |
|  | Sample20 | 1 | 16.44 | 1 | 16.21 | 1 | 16.32 |
|  | Sample21 | 1 | 16.45 | 1 | 16.28 | 1 | 16.33 |
|  | Sample22 | 1 | 17.63 | 1 | 17.73 | 1 | 17.54 |
|  | Sample23 | 1 | 19.77 | 1 | 19.63 | 1 | 19.47 |
|  | Sample24 | 1 | 16.28 | 1 | 16.25 | 1 | 16.11 |
| N/A, not available | | | | | | | |

| Table S7: Detection of PAX1 by modified MSRE - qPCR in cervical squamous cell carcinoma and normal group samples (ABI7500) | | | | | | | | | | | | | | | | |  |
| --- | --- | --- | --- | --- | --- | --- | --- | --- | --- | --- | --- | --- | --- | --- | --- | --- | --- |
|  | 1：2 | | 1：5 | | 1：10 | | 1：20 | | 1：50 | | 1：100 | | 1：1000 | | 1：10000 | |  |
| Sample Name | Instrument  /ABI7500 | Target Ct | Instrument  /ABI7500 | Target Ct | Instrument  /ABI7500 | Target Ct | Instrument  /ABI7500 | Target Ct | Instrument  /ABI75000 | Target Ct | Instrument  /ABI7500 | Target Ct | Instrument/ABI7500 | Target Ct | Instrument/ABI7500 | Target Ct | |
| No. |  |  |  |  |  |  |  |  |  |  |  |  |  |  |  |  | |
| 1 | 1 | 4.98 | 1 | 6.56 | 1 | 8.20 | 1 | 9.43 | 1 | 11.32 | 1 | 13.78 | 1 | 21.43 | 1 | 33.47 | |
| 2 | 1 | 4.61 | 1 | 6.40 | 1 | 7.75 | 1 | 9.39 | 1 | 11.31 | 1 | 12.76 | 1 | 19.52 | 1 | 33.48 | |
| 3 | 1 | 4.39 | 1 | 6.26 | 1 | 7.79 | 1 | 9.43 | 1 | 11.17 | 1 | 12.86 | 1 | 19.04 | 1 | 28.25 | |
| 4 | 1 | 4.77 | 1 | 5.89 | 1 | 7.49 | 1 | 9.57 | 1 | 11.32 | 1 | 12.96 | 1 | 20.20 | 1 | 29.41 | |
| 5 | 1 | 4.51 | 1 | 6.21 | 1 | 7.36 | 1 | 8.99 | 1 | 11.74 | 1 | 16.00 | 1 | 23.58 | 1 | 28.21 | |
| 6 | 1 | 5.25 | 1 | 6.64 | 1 | 9.27 | 1 | N/A | 1 | N/A | 1 | 13.69 | 1 | 26.88 | 1 | 30.19 | |
| 7 | 1 | 4.78 | 1 | 6.13 | 1 | 7.72 | 1 | 9.37 | 1 | 11.44 | 1 | 13.04 | 1 | 20.56 | 1 | 33.02 | |
| 8 | 1 | 5.28 | 1 | 6.99 | 1 | 8.51 | 1 | 10.63 | 1 | 12.00 | 1 | 14.30 | 1 | 22.18 | 1 | 33.02 | |
| 9 | 1 | N/A | 1 | N/A | 1 | 8.85 | 1 | 10.84 | 1 | 12.82 | 1 | 15.01 | 1 | 21.43 | 1 | N/A | |
| 10 | 1 | N/A | 1 | N/A | 1 | N/A | 1 | N/A | 1 | 11.32 | 1 | N/A | 1 | N/A | 1 | 32.36 | |
| 11 | 1 | N/A | 1 | N/A | 1 | N/A | 1 | N/A | 1 | 11.31 | 1 | N/A | 1 | N/A | 1 | 32.25 | |
| 12 | 1 | N/A | 1 | N/A | 1 | N/A | 1 | N/A | 1 | 11.17 | 1 | N/A | 1 | N/A | 1 | 29.79 | |
| 13 | 1 | N/A | 1 | N/A | 1 | N/A | 1 | N/A | 1 | N/A | 1 | N/A | 1 | N/A | 1 | N/A | |
| 14 | 1 | N/A | 1 | 7.49 | 1 | N/A | 1 | N/A | 1 | N/A | 1 | N/A | 1 | N/A | 1 | N/A | |
| 15 | 1 | N/A | 1 | N/A | 1 | N/A | 1 | N/A | 1 | N/A | 1 | N/A | 1 | N/A | 1 | N/A | |
| 16 | 1 | N/A | 1 | N/A | 1 | N/A | 1 | N/A | 1 | N/A | 1 | N/A | 1 | N/A | 1 | 30.42 | |
| 17 | 1 | N/A | 1 | N/A | 1 | N/A | 1 | N/A | 1 | N/A | 1 | N/A | 1 | N/A | 1 | 29.27 | |
| 18 | 1 | N/A | 1 | N/A | 1 | N/A | 1 | N/A | 1 | N/A | 1 | N/A | 1 | N/A | 1 | 30.93 | |
| 19 | 1 | N/A | 1 | N/A | 1 | N/A | 1 | N/A | 1 | N/A | 1 | N/A | 1 | N/A | 1 | 29.44 | |
| 20 | 1 | N/A | 1 | N/A | 1 | N/A | 1 | N/A | 1 | N/A | 1 | N/A | 1 | N/A | 1 | 29.27 | |
| 21 | 1 | N/A | 1 | N/A | 1 | N/A | 1 | N/A | 1 | N/A | 1 | N/A | 1 | N/A | 1 | 31.00 | |
| 22 | 1 | N/A | 1 | N/A | 1 | N/A | 1 | N/A | 1 | N/A | 1 | N/A | 1 | N/A | 1 | 31.98 | |
| 23 | 1 | N/A | 1 | N/A | 1 | N/A | 1 | N/A | 1 | N/A | 1 | N/A | 1 | N/A | 1 | 32.80 | |
| 24 | 1 | N/A | 1 | N/A | 1 | N/A | 1 | N/A | 1 | N/A | 1 | N/A | 1 | N/A | 1 | 32.47 | |
| Nor.1 | 1 | N/A | 1 | N/A | 1 | 12.38 | 1 | 13.86 | 1 | 16.44 | 1 | 19.58 | 1 | 29.99 | 1 | 33.41 | |
| Nor.2 | 1 | N/A | 1 | N/A | 1 | N/A | 1 | N/A | 1 | N/A | 1 | N/A | 1 | N/A | 1 | N/A | |
| Nor.3 | 1 | 8.01 | 1 | 10.02 | 1 | 12.78 | 1 | 13.83 | 1 | 15.88 | 1 | 17.58 | 1 | 26.71 | 1 | 30.13 | |
| Nor.4 | 1 | 6.89 | 1 | 9.18 | 1 | 14.56 | 1 | 19.62 | 1 | 18.99 | 1 | N/A | 1 | 29.52 | 1 | 31.09 | |
| Nor.9 | 1 | N/A | 1 | N/A | 1 | 9.98 | 1 | 12.82 | 1 | 14.97 | 1 | 15.51 | 1 | 31.09 | 1 | 36.02 | |
| Nor.10 | 1 | N/A | 1 | N/A | 1 | 9.64 | 1 | 12.07 | 1 | 14.96 | 1 | 15.28 | 1 | 27.96 | 1 | 35.09 | |
| No.11 | 1 | N/A | 1 | N/A | 1 | 9.60 | 1 | 11.67 | 1 | 13.42 | 1 | 15.01 | 1 | 22.95 | 1 | 31.09 | |
| N/A, not available （Amplification was detectable after dilution but not at high concentration, suggesting PCR inhibition due to excessive template concentration.） | | | | | | | | | | | | | | | | | |

| Table S8 :Table of ROC analysis for determining the cutoff value of methylation - positive sites that separate CESC* from Normal | | | |
| --- | --- | --- | --- |
| Hypermetnylation Gene of CESC | 1 - specificity | sensitivity | sensitivity - (1 - specificity) |
| SOX1 | 0.08 | 0.66 | 0.58 |
| RXFP3-L1 | 0.09 | 0.66 | 0.57 |
| RXFP3-L2 | 0.09 | 0.65 | 0.56 |
| ZNF671 | 0.09 | 0.26 | 0.17 |
| *CESC denotes highly-methylated cervical cancer. | | | |

| Table S9 : Results of the evaluation of primers and probes specificity (pre- and post-modification of the MSRE-qPCR method)（SLAN-96S) | | | | | |
| --- | --- | --- | --- | --- | --- |
|  | | Modified MSRE-qPCR | | MSRE-qPCR | |
| Gene | Sample Name | Instrument/SLAN | Target Ct(Mean) | Instrument/SLAN | Target Ct(Mean) |
| PAX1 | Sample |  |  |  |  |
|  | Sample1 | 2 | 15.33 | 2 | 20.86 |
|  | Sample2 | 2 | 13.29 | 2 | 21.73 |
|  | Sample3 | 2 | 19.82 | 2 | N/A |
|  | Sample4 | 2 | 20.27 | 2 | 30.59 |
|  | Sample5 | 2 | 25.61 | 2 | 31.53 |
|  | Sample6 | 2 | 24.21 | 2 | N/A |
|  | Sample7 | 2 | 12.65 | 2 | 34.71 |
|  | Sample8 | 2 | 26.53 | 2 | 31.30 |
|  | Sample9 | 2 | N/A | 2 | 20.86 |
|  | Sample10 | 2 | N/A | 2 | 21.73 |
|  | Sample11 | 2 | N/A | 2 | N/A |
|  | Sample12 | 2 | N/A | 2 | 30.59 |
|  | Sample13 | 2 | N/A | 2 | 31.53 |
|  | Sample14 | 2 | N/A | 2 | N/A |
|  | Sample15 | 2 | N/A | 2 | 34.71 |
|  | Sample16 | 2 | N/A | 2 | 31.30 |
| SOX1 | Sample |  |  |  |  |
|  | Sample1 | 2 | 14.85 | 2 | 15.68 |
|  | Sample2 | 2 | 14.21 | 2 | 15.27 |
|  | Sample3 | 2 | 15.41 | 2 | N/A |
|  | Sample4 | 2 | 18.15 | 2 | N/A |
|  | Sample5 | 2 | N/A | 2 | 21.48 |
|  | Sample6 | 2 | N/A | 2 | 18.48 |
|  | Sample7 | 2 | N/A | 2 | 22.05 |
|  | Sample8 | 2 | N/A | 2 | N/A |
|  | Sample9 | 2 | 22.02 | 2 | 42.57 |
|  | Sample10 | 2 | 21.55 | 2 | 44.83 |
|  | Sample11 | 2 | 13.49 | 2 | 15.98 |
|  | Sample12 | 2 | 13.44 | 2 | N/A |
|  | Sample13 | 2 | 12.26 | 2 | 37.37 |
|  | Sample14 | 2 | 11.96 | 2 | 39.92 |
|  | Sample15 | 2 | 13.28 | 2 | 38.65 |
|  | Sample16 | 2 | 11.63 | 2 | 12.30 |
| ZNF671 | Sample |  |  |  |  |
|  | Sample1 | 2 | 11.64 | 2 | 11.18 |
|  | Sample2 | 2 | 12.97 | 2 | 15.37 |
|  | Sample3 | 2 | N/A | 2 | 20.37 |
|  | Sample4 | 2 | 17.57 | 2 | N/A |
|  | Sample5 | 2 | 14.26 | 2 | N/A |
|  | Sample6 | 2 | 14.40 | 2 | 17.08 |
|  | Sample7 | 2 | 14.61 | 2 | 19.11 |
|  | Sample8 | 2 | 14.40 | 2 | 20.33 |
|  | Sample9 | 2 | 20.16 | 2 | 12.51 |
|  | Sample10 | 2 | 19.45 | 2 | 16.00 |
|  | Sample11 | 2 | 13.31 | 2 | 15.95 |
|  | Sample12 | 2 | 14.93 | 2 | 15.27 |
|  | Sample13 | 2 | 14.2 | 2 | 31.39 |
|  | Sample14 | 2 | 11.29 | 2 | 14.04 |
|  | Sample15 | 2 | 16.76 | 2 | 14.4 |
|  | Sample16 | 2 | 13.14 | 2 | 15.89 |
| RXFP3-L1 | Sample |  |  |  |  |
|  | Sample1 | 2 | N/A | 2 | 32.33 |
|  | Sample2 | 2 | 21.02 | 2 | N/A |
|  | Sample3 | 2 | 15.31 | 2 | N/A |
|  | Sample4 | 2 | 16.35 | 2 | 15.82 |
|  | Sample5 | 2 | N/A | 2 | 16.66 |
|  | Sample6 | 2 | 17.20 | 2 | N/A |
|  | Sample7 | 2 | N/A | 2 | 23.87 |
|  | Sample8 | 2 | 16.26 | 2 | 19.91 |
|  | Sample9 | 2 | 15.16 | 2 | N/A |
|  | Sample10 | 2 | 16.77 | 2 | 17.10 |
|  | Sample11 | 2 | 13.52 | 2 | 13.58 |
|  | Sample12 | 2 | 21.21 | 2 | 21.66 |
|  | Sample13 | 2 | 16.00 | 2 | 16.48 |
|  | Sample14 | 2 | 16.97 | 2 | 16.99 |
|  | Sample15 | 2 | 16.31 | 2 | 16.33 |
|  | Sample16 | 2 | 15.30 | 2 | 15.24 |
| RXFP3-L2 | Sample |  |  |  |  |
|  | Sample1 | 2 | 9.13 | 2 | 11.63 |
|  | Sample2 | 2 | 8.92 | 2 | 14.48 |
|  | Sample3 | 2 | 12.15 | 2 | 14.36 |
|  | Sample4 | 2 | 7.87 | 2 | 12.74 |
|  | Sample5 | 2 | 16.44 | 2 | 17.55 |
|  | Sample6 | 2 | 15.92 | 2 | 17.96 |
|  | Sample7 | 2 | 16.38 | 2 | 15.87 |
|  | Sample8 | 2 | 16.94 | 2 | 13.91 |
|  | Sample9 | 2 | 16.47 | 2 | 12.24 |
|  | Sample10 | 2 | 16.46 | 2 | 13.68 |
|  | Sample11 | 2 | 11.33 | 2 | 11.29 |
|  | Sample12 | 2 | 13.08 | 2 | 12.54 |
|  | Sample13 | 2 | 13.33 | 2 | 14.82 |
|  | Sample14 | 2 | 10.90 | 2 | 13.50 |
|  | Sample15 | 2 | 12.69 | 2 | 16.12 |
|  | Sample16 | 2 | 11.13 | 2 | 10.54 |
| Note: N/A, not available (1. Not detected after 45 cycles. 2. Amplification was detectable after dilution but not at high concentration.) | | | | | |

| Table S10 : Results of the evaluation of cfDNA and tiDNA for improved MSRE - qPCR detection (ABI 7500) | | | | | |
| --- | --- | --- | --- | --- | --- |
|  | | cfDNA | | tiDNA | |
| Gene | Sample Name | Instrument/ABI7500 | Target Ct(Mean) | Instrument/ABI7500 | Target Ct(Mean) |
| PAX1 | Sample |  |  |  |  |
|  | Sample1 | 1 | 15.33 | 1 | 10.22 |
|  | Sample2 | 1 | 13.29 | 1 | N/A |
|  | Sample3 | 1 | 19.82 | 1 | 10.30 |
|  | Sample4 | 1 | 20.27 | 1 | N/A |
|  | Sample5 | 1 | 25.61 | 1 | 10.19 |
|  | Sample6 | 1 | 24.21 | 1 | 10.28 |
|  | Sample7 | 1 | 12.65 | 1 | 10.21 |
|  | Sample8 | 1 | N/A | 1 | 10.68 |
|  | Sample9 | 1 | N/A | 1 | 10.95 |
|  | Sample10 | 1 | N/A | 1 | 10.52 |
|  | Sample11 | 1 | N/A | 1 | 10.09 |
|  | Sample12 | 1 | N/A | 1 | 10.04 |
|  | Sample13 | 1 | N/A | 1 | 10.03 |
|  | Sample14 | 1 | N/A | 1 | 10.01 |
|  | Sample15 | 1 | N/A | 1 | 10.22 |
|  | Sample16 | 1 | N/A | 1 | 10.01 |
|  | Sample17 | 1 | N/A | 1 | 10.15 |
|  | Sample18 | 1 | N/A | 1 | 10.17 |
|  | Sample19 | 1 | N/A | 1 | 10.10 |
|  | Sample20 | 1 | N/A | 1 | N/A |
|  | Sample21 | 1 | 24.16 | 1 | 10.10 |
|  | Sample22 | 1 | N/A | 1 | 10.10 |
|  | Sample23 | 1 | N/A | 1 | 10.15 |
|  | Sample24 | 1 | N/A | 1 | 10.08 |
| SOX1 | Sample |  |  |  |  |
|  | Sample1 | 1 | 4.62 | 1 | 14.01 |
|  | Sample2 | 1 | 4.60 | 1 | N/A |
|  | Sample3 | 1 | 4.61 | 1 | 13.55 |
|  | Sample4 | 1 | 4.62 | 1 | N/A |
|  | Sample5 | 1 | 4.81 | 1 | 13.73 |
|  | Sample6 | 1 | 5.09 | 1 | 11.49 |
|  | Sample7 | 1 | 4.56 | 1 | 14.89 |
|  | Sample8 | 1 | 4.74 | 1 | 17.35 |
|  | Sample9 | 1 | 5.69 | 1 | 17.20 |
|  | Sample10 | 1 | 5.25 | 1 | N/A |
|  | Sample11 | 1 | 13.49 | 1 | 16.17 |
|  | Sample12 | 1 | 13.44 | 1 | 14.66 |
|  | Sample13 | 1 | 12.26 | 1 | 18.54 |
|  | Sample14 | 1 | 11.96 | 1 | 16.92 |
|  | Sample15 | 1 | 13.28 | 1 | 4.97 |
|  | Sample16 | 1 | 11.63 | 1 | 5.18 |
|  | Sample17 | 1 | 16.35 | 1 | 4.965 |
|  | Sample18 | 1 | 14.50 | 1 | 5.05 |
|  | Sample19 | 1 | 6.14 | 1 | 5.20 |
|  | Sample20 | 1 | 5.15 | 1 | N/A |
|  | Sample21 | 1 | 5.26 | 1 | 4.73 |
|  | Sample22 | 1 | 5.51 | 1 | 5.18 |
|  | Sample23 | 1 | 5.37 | 1 | 4.99 |
|  | Sample24 | 1 | 5.06 | 1 | 5.10 |
| ZNF671 | Sample |  |  |  |  |
|  | Sample1 | 1 | 7.61 | 1 | 4.59 |
|  | Sample2 | 1 | 7.50 | 1 | N/A |
|  | Sample3 | 1 | 6.06 | 1 | 4.26 |
|  | Sample4 | 1 | 5.10 | 1 | N/A |
|  | Sample5 | 1 | 6.28 | 1 | 4.36 |
|  | Sample6 | 1 | 5.12 | 1 | 4.28 |
|  | Sample7 | 1 | 7.37 | 1 | 12.03 |
|  | Sample8 | 1 | 4.11 | 1 | 12.46 |
|  | Sample9 | 1 | 8.15 | 1 | N/A |
|  | Sample10 | 1 | 8.19 | 1 | 11.14 |
|  | Sample11 | 1 | 13.31 | 1 | 6.10 |
|  | Sample12 | 1 | 14.93 | 1 | 5.67 |
|  | Sample13 | 1 | 14.20 | 1 | 5.70 |
|  | Sample14 | 1 | 11.29 | 1 | 5.75 |
|  | Sample15 | 1 | 16.76 | 1 | 4.24 |
|  | Sample16 | 1 | 13.14 | 1 | 4.56 |
|  | Sample17 | 1 | 6.14 | 1 | 7.825 |
|  | Sample18 | 1 | 12.09 | 1 | 4.06 |
|  | Sample19 | 1 | 8.29 | 1 | 14.20 |
|  | Sample20 | 1 | 9.40 | 1 | N/A |
|  | Sample21 | 1 | 10.58 | 1 | 13.76 |
|  | Sample22 | 1 | 11.21 | 1 | 12.85 |
|  | Sample23 | 1 | 11.18 | 1 | 13.32 |
|  | Sample24 | 1 | N/A | 1 | 12.87 |
| RXFP3-L1 | Sample |  |  |  |  |
|  | Sample1 | 1 | 10.74 | 1 | 10.51 |
|  | Sample2 | 1 | 10.47 | 1 | N/A |
|  | Sample3 | 1 | 9.93 | 1 | 10.49 |
|  | Sample4 | 1 | 10.27 | 1 | N/A |
|  | Sample5 | 1 | 10.44 | 1 | 10.85 |
|  | Sample6 | 1 | 10.49 | 1 | 10.14 |
|  | Sample7 | 1 | 8.54 | 1 | 15.95 |
|  | Sample8 | 1 | 9.07 | 1 | 15.32 |
|  | Sample9 | 1 | 15.16 | 1 | 14.66 |
|  | Sample10 | 1 | 16.77 | 1 | 13.91 |
|  | Sample11 | 1 | 13.52 | 1 | 14.38 |
|  | Sample12 | 1 | 21.21 | 1 | 14.86 |
|  | Sample13 | 1 | 16.00 | 1 | 13.45 |
|  | Sample14 | 1 | 16.97 | 1 | 13.79 |
|  | Sample15 | 1 | 16.31 | 1 | 12.11 |
|  | Sample16 | 1 | 15.30 | 1 | 6.01 |
|  | Sample17 | 1 | 13.07 | 1 | 9.07 |
|  | Sample18 | 1 | 12.01 | 1 | 5.41 |
|  | Sample19 | 1 | 12.12 | 1 | 18.12 |
|  | Sample20 | 1 | 11.54 | 1 | N/A |
|  | Sample21 | 1 | 12.98 | 1 | 17.79 |
|  | Sample22 | 1 | 14.83 | 1 | 17.58 |
|  | Sample23 | 1 | 15.36 | 1 | 18.23 |
|  | Sample24 | 1 | 14.82 | 1 | 17.20 |
| RXFP3-L2 | Sample |  |  |  |  |
|  | Sample1 | 1 | 5.52 | 1 | 6.82 |
|  | Sample2 | 1 | 5.50 | 1 | N/A |
|  | Sample3 | 1 | 5.16 | 1 | 7.25 |
|  | Sample4 | 1 | 5.31 | 1 | N/A |
|  | Sample5 | 1 | 6.24 | 1 | 8.29 |
|  | Sample6 | 1 | 5.60 | 1 | 7.62 |
|  | Sample7 | 1 | 6.53 | 1 | 7.87 |
|  | Sample8 | 1 | 8.43 | 1 | 8.34 |
|  | Sample9 | 1 | 5.44 | 1 | 7.97 |
|  | Sample10 | 1 | 6.29 | 1 | 7.64 |
|  | Sample11 | 1 | 11.33 | 1 | 5.12 |
|  | Sample12 | 1 | 13.04 | 1 | 5.25 |
|  | Sample13 | 1 | 13.33 | 1 | 5.16 |
|  | Sample14 | 1 | 10.90 | 1 | 5.12 |
|  | Sample15 | 1 | 12.69 | 1 | 5.00 |
|  | Sample16 | 1 | 11.13 | 1 | N/A |
|  | Sample17 | 1 | 12.28 | 1 | 4.55 |
|  | Sample18 | 1 | 10.61 | 1 | 4.35 |
|  | Sample19 | 1 | 9.50 | 1 | 9.91 |
|  | Sample20 | 1 | 7.63 | 1 | N/A |
|  | Sample21 | 1 | 7.62 | 1 | 9.50 |
|  | Sample22 | 1 | 7.07 | 1 | 9.24 |
|  | Sample23 | 1 | 7.42 | 1 | 9.46 |
|  | Sample24 | 1 | N/A | 1 | 9.09 |
|  | Sample20 | 1 | 7.63 | 1 | N/A |
|  | Sample21 | 1 | 7.62 | 1 | 9.50 |
|  | Sample22 | 1 | 7.07 | 1 | 9.24 |
|  | Sample23 | 1 | 7.42 | 1 | 9.46 |
|  | Sample24 | 1 | N/A | 1 | 9.09 |
| Note: N/A, not available (Amplification was detectable after dilution but not at high concentration, suggesting PCR inhibition due to excessive template concentration.) | | | | | |

| Table S11 : MethyLight interpretation and rules of test method results | | | | |
| --- | --- | --- | --- | --- |
| Texas Red  （ACTB） | FAM  （PAX1） | VIC  （SOX1） | CY5  （HAS1） | Interpretation of results |
| Ct≤33.0 | ∆Ct≤6.5 | ∆Ct≤7.0 | ∆Ct≤5.0 | positive |
|  | ∆Ct≤6.5 | ∆Ct≤7.0 | ∆Ct>5.0 |  |
|  | ∆Ct≤6.5 | ∆Ct>7.0 | ∆Ct≤5.0 |  |
|  | ∆Ct≤6.5 | ∆Ct>7.0 | ∆Ct>5.0 |  |
|  | ∆Ct>6.5 | ∆Ct≤7.0 | ∆Ct≤5.0 |  |
|  | ∆Ct>6.5 | ∆Ct≤7.0 | ∆Ct>5.0 |  |
|  | ∆Ct>6.5 | ∆Ct>7.0 | ∆Ct≤5.0 |  |
|  | ∆Ct>6.5 | ∆Ct>7.0 | ∆Ct>5.0 | negtive |
| Ct＞33.0 | - | - | - | - |
| The amplification curve shows no significant logarithmic growth phase or amplification curve | - | - | - |  |
| Note:“-”The value is invalid | | | | |

| Table S12 : MethyLight positive judgment value of test method | | |
| --- | --- | --- |
| Gene | Channel | Positive judgment value |
| ACTB | Texas Red | ∆Ct≤33.0 |
| PAX1 | FAM | ∆Ct≤6.5 |
| SOX1 | VIC | ∆Ct≤7.0 |
| HAS1 | CY5 | ∆Ct≤5.0 |
| Note: ∆Ct=Ct(Target)-Ct(Texas Red) | | |
